# Selective Roles of Vertebrate PCF11 in Premature and Full-Length Transcript Termination

**DOI:** 10.1016/j.molcel.2019.01.027

**Published:** 2019-04-04

**Authors:** Kinga Kamieniarz-Gdula, Michal R. Gdula, Karin Panser, Takayuki Nojima, Joan Monks, Jacek R. Wiśniewski, Joey Riepsaame, Neil Brockdorff, Andrea Pauli, Nick J. Proudfoot

**Affiliations:** 1 Sir William Dunn School of Pathology, University of Oxford, South Parks Road, Oxford OX1 3RE, UK; 2 Department of Biochemistry, University of Oxford, South Parks Road, Oxford OX1 3QU, UK; 3 Research Institute of Molecular Pathology (IMP), Vienna Biocenter (VBC), Campus-Vienna-Biocenter 1, 1030 Vienna, Austria; 4 Biochemical Proteomics Group, Department of Proteomics and Signal Transduction, Max-Planck Institute of Biochemistry, Am Klopferspitz 18, 82152 Martinsried, Germany

**Keywords:** PCF11, transcription termination, alternative polyadenylation, RNA 3′ processing, attenuation, premature termination, regulation of gene expression, autoregulation, human, zebrafish

## Abstract

The pervasive nature of RNA polymerase II (Pol II) transcription requires efficient termination. A key player in this process is the cleavage and polyadenylation (CPA) factor PCF11, which directly binds to the Pol II C-terminal domain and dismantles elongating Pol II from DNA *in vitro*. We demonstrate that PCF11-mediated termination is essential for vertebrate development. A range of genomic analyses, including mNET-seq, 3′ mRNA-seq, chromatin RNA-seq, and ChIP-seq, reveals that PCF11 enhances transcription termination and stimulates early polyadenylation genome-wide. PCF11 binds preferentially between closely spaced genes, where it prevents transcriptional interference and consequent gene downregulation. Notably, PCF11 is sub-stoichiometric to the CPA complex. Low levels of PCF11 are maintained by an auto-regulatory mechanism involving premature termination of its own transcript and are important for normal development. Both in human cell culture and during zebrafish development, PCF11 selectively attenuates the expression of other transcriptional regulators by premature CPA and termination.

## Highlights

• Human PCF11 enhances transcription termination and 3′ end processing, genome-wide
• PCF11 is substoichiometric to CPA complex due to autoregulation of its transcription
• PCF11 stimulates expression of closely spaced genes but attenuates other genes
• PCF11-mediated functions are conserved in vertebrates and essential in development

## Graphical Abstract

**Figure:**
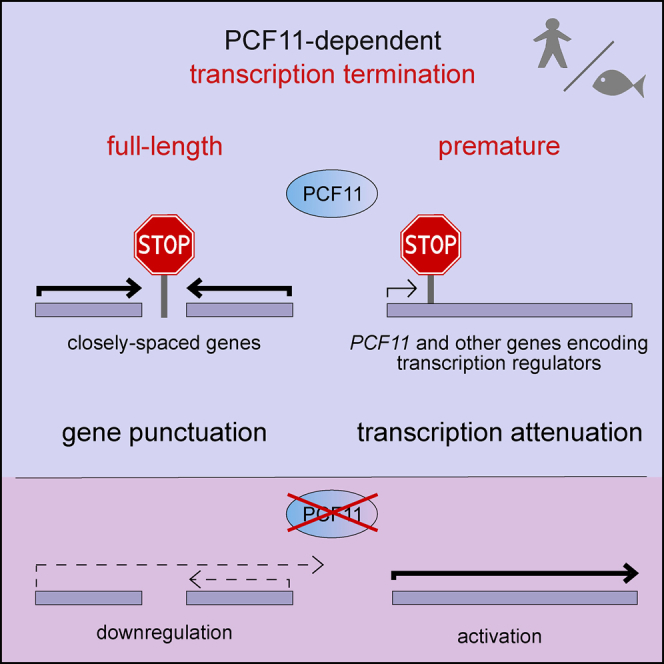

## Teaser

Kamieniarz-Gdula and colleagues demonstrate that vertebrate PCF11 plays selective roles in regulating transcriptional termination and RNA 3′ end processing. It is important for efficient expression of closely spaced genes but in some cases promotes premature termination and gene downregulation. Especially PCF11 transcript levels are kept low by this attenuation mechanism.

## Introduction

RNA polymerase II (Pol II)-mediated transcription involves a cycle of initiation, elongation, and termination. Transcription termination stops RNA synthesis through release of Pol II and RNA from the DNA template. This process is crucial for correct gene expression. First, termination punctuates the ends of transcription units by releasing RNA to fulfill its biological function. Second, it ensures Pol II availability for subsequent rounds of RNA synthesis. Third, it restricts the extent of non-coding (nc) transcription and prevents transcriptional interference between adjacent transcriptional units (Jensen et al., 2013, Porrua et al., 2016, Proudfoot, 2016).

Mechanistically, termination is coupled to 3′ end processing of pre-mRNAs. Pol II becomes termination competent after transcribing a polyadenylation signal (poly(A) signal, typically including the hexamer AAUAAA). This signal in the nascent RNA is recognized by the RNA 3′ processing machinery, which promotes RNA cleavage and polyadenylation 10–30 nucleotides downstream at the polyadenylation site (PAS) (Proudfoot, 2011). Cleavage of the nascent transcript at the PAS is coupled to 5′ > 3′ degradation of the downstream RNA by XRN2, which eventually leads to termination (Porrua et al., 2016, Proudfoot, 2016). In mammalian genomes, Pol II typically continues transcribing thousands of base pairs downstream of the PAS (Nojima et al., 2015, Schwalb et al., 2016). In summary, although the cleavage and polyadenylation (CPA) step occurs at defined locations (PAS), Pol II continues to transcribe the downstream sequences over a wide genomic window.

Most mammalian protein-coding (pc) genes contain multiple alternative poly(A) signals. If more than one site within a transcript is able to support RNA cleavage, distinct RNA 3′ termini can be generated, in a mechanism called alternative polyadenylation (APA) (Tian and Manley, 2017). APA within gene-coding regions can result in truncated polypeptides with diverse functions, as demonstrated for genes encoding calcitonin (Amara et al., 1984) and immunoglobulin heavy chain (Takagaki et al., 1996). Genome-wide studies have demonstrated that 70% of mammalian pc genes generate mRNA with alternative 3′ ends, often differing in their 3′ UTR. Alternative 3′ UTRs can confer different functions and stability to mRNA depending on the presence of AU-rich elements and binding sites for miRNA and RNA-binding proteins (Tian and Manley, 2017).

3′ ends of mammalian mRNA are processed by a large CPA complex, which includes cleavage and polyadenylation specificity factor (CPSF), cleavage stimulation factor (CstF), and cleavage factors I and II (CFIm and CFIIm), each consisting of multiple subunits (Shi and Manley, 2015). CFIIm contains two proteins, PCF11 and CLP1, and—unlike other CPA factors—interacts only weakly and/or transiently with the complex (Shi et al., 2009). Most CPA proteins participate in defined steps, such as the cleavage reaction or recognition of specific RNA motifs. In contrast, PCF11 is critical not only for 3′ processing (Amrani et al., 1997, de Vries et al., 2000, Gross and Moore, 2001) but also for transcription termination (Zhang et al., 2005, Zhang and Gilmour, 2006, West and Proudfoot, 2008) and links transcription with mRNA export (Johnson et al., 2009, Volanakis et al., 2017). In yeast, the 3′ end-processing and termination activities of PCF11 are provided by distinct PCF11 domains and can be functionally uncoupled (Sadowski et al., 2003). PCF11 is able to bind to the C-terminal domain (CTD) of the largest subunit of Pol II via its conserved CTD interaction domain (CID) (Barillà et al., 2001, Meinhart and Cramer, 2004, Kecman et al., 2018). The CID-CTD interaction dismantles elongation complexes *in vitro* (Zhang et al., 2005, Zhang and Gilmour, 2006) and is required for normal Pol II CTD serine 2 phosphorylation (S2ph) levels in yeast (Grzechnik et al., 2015). PCF11 CID also displays RNA binding activity, and a competition between RNA and CTD binding by the CID has been proposed to mediate Pol II disengagement (Zhang et al., 2005, Hollingworth et al., 2006). A second RNA-binding domain is present in the C-terminal part of the protein (Schäfer et al., 2018).

Although PCF11 is a key factor acting at the intersection of several nuclear processes, it has mainly been studied in yeast, with little knowledge of its function in vertebrates. However, three independent pan-cancer screens for cancer driver mutations have recently identified recurrent mutations in *PCF11*, in particular within the promoter region (Hornshøj et al., 2018, Kuipers et al., 2018, Rheinbay et al., 2017). Moreover, PCF11 expression levels are predictive of clinical outcomes of neuroblastoma patients (Ogorodnikov et al., 2018), suggesting that PCF11 has relevance to human pathology. We address here the genome-wide role of PCF11 in vertebrate gene expression.

## Results

### PCF11 Enhances Transcription Termination and CPA Genome-wide

PCF11 was depleted using a pool of four small interfering RNAs (siRNAs) optimized for knockdown duration and siRNA dosage (Figures S1A and S1B). Pol II binding was assessed by chromatin immunoprecipitation sequencing (ChIP-seq) for total Pol II (using N20 antibody). Transcriptional output was also measured by analysis of chromatin-bound RNA (chrRNA), enriched for nascent transcripts. Finally, mammalian native elongating transcript sequencing (mNET-seq) (Nojima et al., 2015) was used to assay nascent transcripts associated with threonine 4 phosphorylated (T4ph) Pol II CTD, which is specific to the termination region (Heidemann et al., 2013, Schlackow et al., 2017).

**Figure 1:**
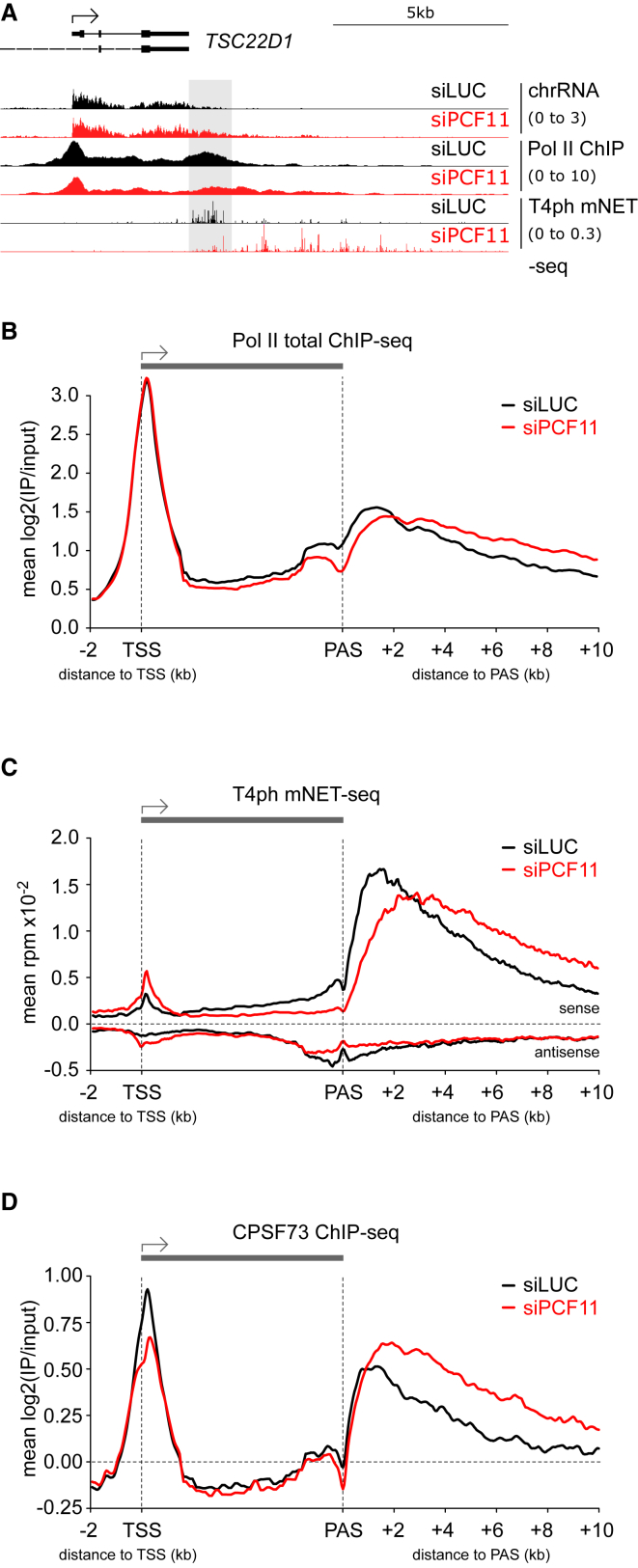
PCF11 Enhances Transcription Termination Genome-wide (A) Genomic profile of *TSC22D1*. Gray shading: termination window in control cells (siLUC, black). PCF11 depletion (siPCF11) is depicted in red. For chrRNA-seq and mNET-seq only sense strand is shown; ChIP is not strand specific. In all profiles, numbers in brackets indicate the viewing range (rpm). (B–D) Metagene analysis of total Pol II ChIP-seq (B), T4ph mNET-seq (C), and CPSF73 ChIP-seq (D) in cells ± siPCF11 on pc genes >5 kb long with PAS separated by >6 kb from the nearest gene on the same strand (n = 8,389).

PCF11 depletion led to transcriptional readthrough beyond usual end sites (Figures 1A–1C, S1C, and S1D). While many genes show Pol II accumulation downstream of the PAS, PCF11 depletion shifted and decreased this Pol II pausing (Figures 1A–1C), both indicative of defective termination. While all assays consistently showed delayed termination in PCF11-depleted conditions, T4ph mNET-seq provided the most specific detection of transcriptional termination. PCF11-depletion-induced termination delay was widespread but only resulted in a shift in the termination window rather than a complete failure to terminate. Downregulation of other human CPA and termination factors in previous studies led to a similar termination shift, suggesting the existence of uncharacterized failsafe termination mechanisms in mammals (Fong et al., 2015, Nojima et al., 2015, Schlackow et al., 2017).

Delayed termination upon PCF11 depletion could be due to a concomitant loss of CPA complex association with termination regions. To test this, we performed ChIP-seq for CPSF73, which is the CPA subunit responsible for pre-mRNA 3′ cleavage. Unexpectedly, PCF11 depletion resulted in increased and 3′ extended CPSF73 signal at gene 3′ ends (Figures 1D and S1E), consistent with prolonged CPSF73 binding to chromatin. Therefore, PCF11 may be unnecessary for CPA complex binding to PAS-proximal regions but rather increase CPA efficiency. We conclude that human PCF11 enhances genome-wide CPA and transcription termination.

### PCF11-Mediated Termination Enhancement Occurs Independently of APA

**Figure 2:**
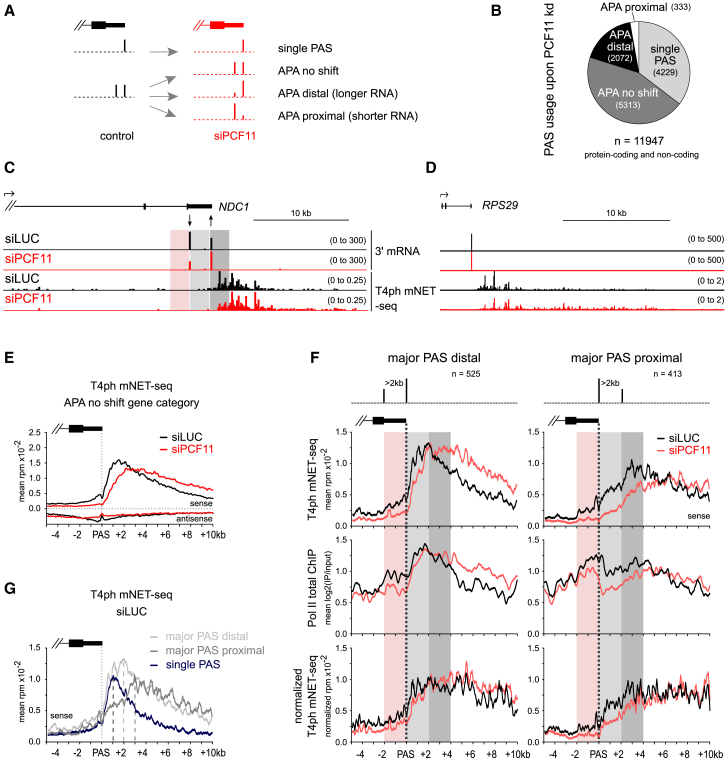
PCF11-Mediated Termination Enhancement Occurs Independently of PAS Selection (A) Schematic of gene type based on APA changes ± siPCF11. (B) Pie chart of PAS usage in cells depleted of PCF11 based on DEXseq analysis (padj < 0.05). (C–D) Genomic profiles of *NDC1* (C) and *RPS29* (D). Arrows indicate significant APA upon PCF11 depletion (DEXseq padj < 0.05; Figure S2B). (E–G) Meta-gene profiles of T4ph mNET-seq signal around major PAS on pc genes. Vertical dotted line: position of the major PAS. (E) Multiple PAS-containing genes without significant change in PAS usage (APA no shift); meta-profiles for other APA gene categories shown in Figure S2E. (F) Genes with two strongest PASs of comparable signal and separated by >2 kb (n = 938) were divided into two sets: those with a major distal (left panels) or proximal (right panels) PAS. Schemes of the PAS positioning are shown on top. Top panels: T4ph mNET-seq; middle panels: Pol II total ChIP-seq; bottom panel: T4ph mNET-seq normalized to Pol II total. Red shading highlights region 2 kb upstream of major PAS, light gray 2 kb downstream, and dark gray 2–4 kb downstream of major PAS (also in C). (G) T4ph mNET-seq profiles in control cells (siLUC) for the indicated gene categories. Vertical dashed lines highlight corresponding T4ph mNET-seq signal maxima.

PCF11 not only affects CPA and termination, but also regulates APA (Li et al., 2015). Readthrough transcription upon PCF11 depletion could be due to either a termination defect or a shift toward distal PAS usage. We therefore determined active PAS usage by sequencing the 3′ ends of nuclear polyadenylated RNA (3′ mRNA sequencing [mRNA-seq]) from control and PCF11-depleted cells. A set of 11,947 pc and nc genes, with PAS at least 6 kb away from the downstream gene on the same strand was selected. PCF11 depletion caused a shift to distal PAS usage for 17% of genes, while only 2.8% revealed a proximal shift, indicating that PCF11 favors proximal PAS usage in human cells (Figures 2A–2C, S2A, and S2B). This effect was more pronounced for pc (22% distal and 3% proximal shifts) than nc genes, of which only 4.4% showed PAS shift (Figure S2A). Genes undergoing APA changes upon PCF11 knockdown had overall higher expression levels than genes where no shift occurred (Figure S2C). Analysis of the 3′ mRNA-seq data also revealed global gene downregulation upon PCF11 depletion (Figure S2D). Most genes with significantly altered expression upon PCF11 knockdown showed no shift in APA usage, suggesting that differential PAS usage is not the major cause of siPCF11-induced gene deregulation. Importantly, the termination loss following PCF11 depletion often occurred without a distal APA shift, and the termination window shifted downstream in all four categories of PAS usage (Figures 2D, 2E, and S2E). We conclude that readthrough transcription upon PCF11 depletion is a hallmark of delayed termination, and not generally due to differential PAS selection.

Intriguingly, we observed numerous genes with well-separated PAS but no T4ph mNET-seq signal associated with the proximal PAS (Figures 2C, S2F, and S2G, light gray shading). To determine whether this is a general trend, we selected a subset of 938 pc APA genes where the two strongest PAS differed no more than 2-fold and were separated by at least 2 kb. If the strongest of the two PAS was distal, the gene was classified as “major PAS distal,” and otherwise as “major PAS proximal” (Figure 2F). The first type showed a sharp increase in T4ph mNET-seq signal after the PAS, matching higher Pol II density in the same region. The second type showed decreased Pol II density and a more gradual increase in T4ph mNET-seq signal downstream of the PAS. PCF11 depletion caused delayed termination for both types (Figure 2F). In control cells, the highest T4ph mNET-seq signal occurred on average 2 kb downstream of the PAS for genes with a major distal PAS and 3 kb for genes with a major proximal PAS, as compared to 1 kb for genes with only 1 PAS (Figure 2G). When normalized to Pol II density, the T4ph mNET-seq signal reached a plateau within 0.5 kb from the PAS for major PAS distal genes, and after 2.5 kb for major PAS proximal genes (Figure 2F, bottom panels). In conclusion, T4ph mNET-seq is more closely associated with distal PAS. This supports the view that CPA and termination might be uncoupled and is consistent with PAS choice occurring in favor of proximal PAS even after the distal PAS has been transcribed (Zhu et al., 2018). Future work involving long-read sequencing will verify whether transcription generally terminates downstream of the distal PAS. We predict that PCF11 plays a specific role in enhancing transcription termination independently of APA selection.

### Genomic Binding Pattern of PCF11

**Figure 3:**
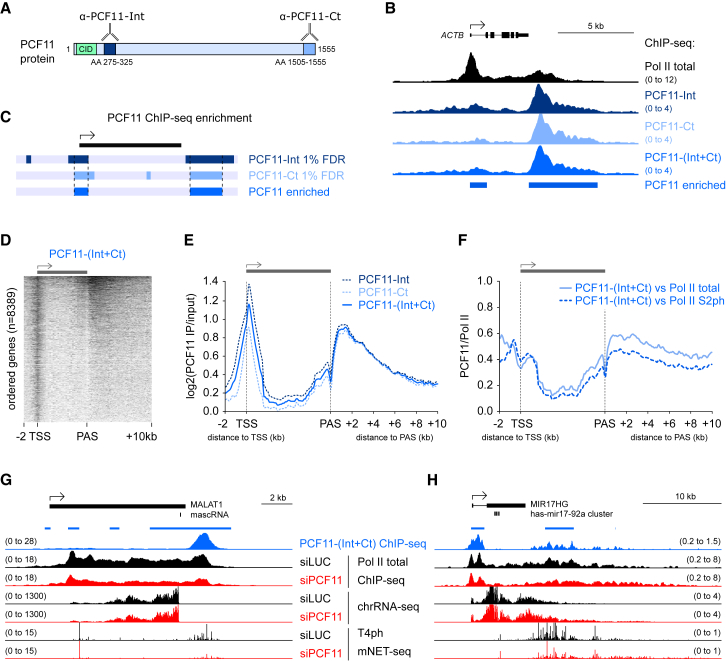
Genomic Pattern of PCF11 Binding (A) Epitopes recognized by PCF11 antibodies; α-PCF11-Int binds an Internal peptide, and α-PCF11-Ct the C terminus. Amino acid (AA) numbering corresponds to the main human PCF11 isoform NP_056969.2. (B) Genomic profile of PCF11 binding to *ACTB*. PCF11-(Int+Ct) corresponds to merged antibody profiles. Blue bars below PCF11 ChIP-seq signal indicate PCF11 enrichment. (C) Enrichment definition: regions with ChIP-seq signal significantly above background for both PCF11 antibodies were considered PCF11-enriched. (D) Heatmap of PCF11-(Int+Ct) ChIP-seq signal (log_2_IP/input) across pc genes ranked from highest to lowest PCF11 signal. (E and F) Meta-gene analysis of PCF11 binding on pc genes (n = 8,389). Plotted is PCF11 ChIP-seq signal relative to input (E) and Pol II (F). PCF11 and Pol II signals were calculated as log_2_(IP/input). (G and H) Genomic profiles showing PCF11 binding and activities on *MALAT1* (G) and *MIR17HG* (H).

We next analyzed the genomic binding profile of PCF11 by ChIP-seq using two different antibodies (Figures 3A, S3A, and S3B), targeting a C-terminal epitope (PCF11-Ct) or internal epitope (PCF11-Int). Both gave similar ChIP-seq profiles (Figures S3C–S3E), so merged signals are also shown (PCF11-(Int+Ct), Figure 3B). We only considered regions significantly above background for both antibodies (1% false discovery rate [FDR]) as PCF11 enriched (Figure 3C).

Consistent with a role in termination, prominent PCF11 binding occurred at gene 3′ ends (Figures 3B, 3D, S3D, and S3E). However, transcription start site (TSS)-proximal enrichment was more frequent (Figures 3D and S3F), leading to a global binding profile at both gene ends (Figure 3E). Upon PCF11 depletion, T4ph mNET-seq signal was not only altered at the 3′ end, but additionally increased in both sense and antisense direction at the TSS (Figure 1C). TSS-associated nc-transcription units, unlike pc genes, are uniformly marked by Pol II T4ph (Schlackow et al., 2017); therefore, the observed increased levels of TSS-associated T4ph mNET-seq are indicative of increased transcription upon PCF11 depletion. This is in line with the previously published role of termination factors in restricting non-productive RNA synthesis at the TSS (Nojima et al., 2015). The pattern of PCF11 binding across genes relative to Pol II (Figures 3F and S3F) suggests that PCF11 does not consistently travel with elongating Pol II from promoter to PAS, although it could transiently interact with Pol II across the gene body.

Even though CPA-dependent pc genes are major targets of PCF11 binding, PCF11 was also detected on transcription units using alternative 3′ processing mechanisms (Figures S3G, 3G, and 3H). Thus the 3′ end of RNase P-processed *MALAT1* is one of the top loci enriched for PCF11. PCF11 depletion did not affect its transcription but decreased Pol II and T4ph mNET-seq signals (Figure 3G). Most other non-canonical PCF11 targets showed no readthrough transcription upon siPCF11, except for microprocessor-dependent long non-coding (lncRNA) microRNA host genes (Dhir et al., 2015) like *MIR17HG* (Figure 3H). PCF11 binding to CPA-independent genes could point to non-canonical functions for PCF11 and CPA factors on these transcripts, or a hybrid mechanism where alternative 3′ processing pathways are used in parallel.

### Closely Spaced Genes Are PCF11 Dependent

**Figure 4:**
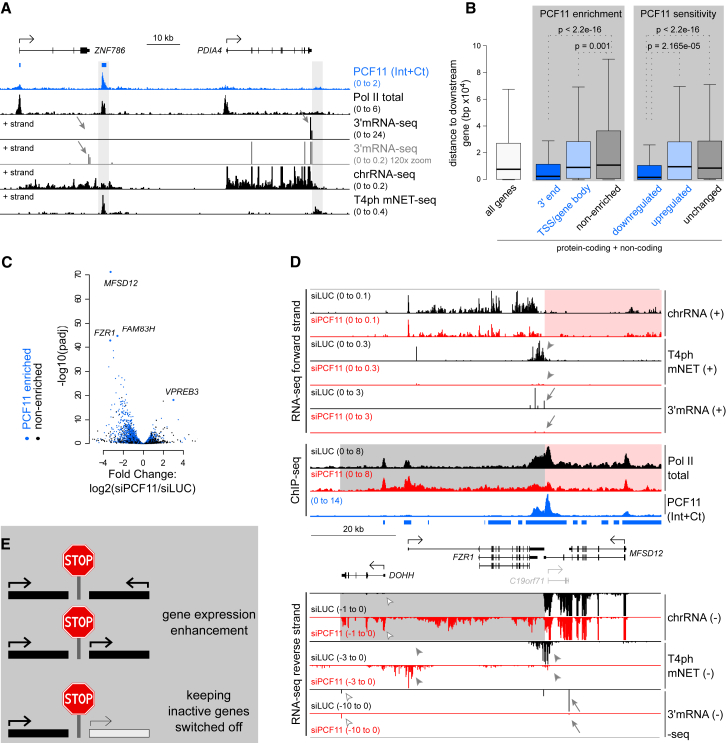
Closely Spaced Genes Are PCF11 Dependent (A) Genomic profile of *ZNF786/PDIA4*. Blue bars indicate PCF11 enrichment. Termination regions are shaded. 3′ mRNA-seq data are shown at two viewing ranges. (B) Boxplot of distances between gene PAS to their nearest gene downstream. Statistical significance was determined using Mann-Whitney test. In all boxplot figures, the thick horizontal line marks median and the upper and lower limits of the box the 1^st^ and 3^rd^ quartile. (C) Volcano plot showing differential expression ± siPCF11. Blue dots correspond to PCF11-enriched genes, and black dots correspond to non-enriched genes. Most significantly deregulated genes are indicated. (D) Genomic profile of the *FZR1/MFSD12* locus. Data from the + strand and non-strand specific data are shown above the locus; data from the – strand are below it. Gray shading: readthrough of *MFSD12*, red shading: lack of detectable readthrough from *FZR1*. *FZR1* is less active (10× zoomed in viewing range). Blue bars: PCF11 enrichment; arrows: gene downregulation; filled arrowheads: alterations in T4ph mNET-seq signal; empty arrowheads: upregulation of *DOHH* due to readthrough from *MFSD12*. (E) Model of PCF11 role in enhancing gene expression of closely spaced genes and isolating inactive genes from upstream tandem active genes.

Although PCF11 binding was detectable on both pc and nc genes, only 54% of tested polyadenylated pc genes had significant PCF11 enrichment (4,516/8,389), of which 47% (2,140) showed enrichment in the 3′ end region. PCF11-enriched genes were globally more highly expressed compared to non-enriched genes (Figure S4A). However, PCF11 enrichment occurred at some silent loci, and, vice versa, some highly expressed genes had no enrichment. For example, PCF11 was enriched at the *ZNF786* but not *PDIA4* termination region (Figure 4A), although the latter is transcriptionally more active (chrRNA-seq) and produces >200-fold more polyadenylated nuclear mRNA (3′ mRNA-seq). *PDIA4* showed a high exonic/intronic chrRNA ratio, implying fast splicing, and had no Pol II accumulation downstream of the PAS. We hypothesize that rapidly processed genes may be bound by PCF11 only transiently.

Visual inspection of PCF11 ChIP-seq data revealed high PCF11 levels between closely spaced genes (Figures S3D and S3E). Accordingly, genes with 3′ PCF11-enrichment showed 4-fold lower spacing compared to non-enriched genes (Figures 4B and S4B). We hypothesize that PCF11 enrichment between closely spaced transcription units prevents transcriptional interference between adjacent genes. Supporting this view, genes significantly downregulated by PCF11 depletion were 5-fold more closely spaced than PCF11-insensitive genes (Figure 4B).

Two of the three most downregulated genes, *MFSD12* and *FZR1*, are convergent neighbors (Figures 4C and 4D). PCF11 showed enrichment over a large part of the locus, especially pronounced between them (Figure 4D). Strong readthrough transcription of the more highly transcribed *MFSD12* gene was evident. Furthermore, T4ph mNET-seq signal was abrogated for *FZR1* and shifted >20 kb for *MFSD12*. This contrasts with isolated genes, where termination is typically shifted only mildly (compare with Figures 1A, 2C, and 2D). Surprisingly, while PCF11 depletion abrogated formation of polyadenylated mRNA products for both genes, transcription, as measured by chrRNA-seq and Pol II ChIP-seq levels, was only mildly decreased (Figure 4D). This suggests that loss of mRNA in the *MFSD12*/*FZR1* locus is not due to transcriptional inhibition but rather a failure of 3′ processing accompanying the severe termination defect. Extensive transcriptional interference can also affect the downstream gene of transcription tandem units, such as *TIMM13* (Figure S4C). Notably, the top two deregulated genes by siPCF11, *MFSD12* and *FAM83H*, showed loss of mRNA in the absence of detectable readthrough transcription from the lower or not expressed close-by genes—*FZR1* and *MAPK15* (Figures 4D and S4D). We speculate that the presence of a close-by downstream gene, even if inactive, does not allow fail-safe termination mechanisms to compensate for PCF11 downregulation. This leads to a failure in both 3′ end processing and termination.

Although PCF11 depletion causes global gene downregulation, some genes were upregulated (Figure 4C, log_2_(siPCF11/siLUC) >0). Visual inspection of these genes (e.g., top upregulated *VPREB3*) revealed that upon PCF11 depletion many were invaded by readthrough transcription from an upstream tandem gene (Figure S4E, see also *DOHH* in Figure 4D). Such fused transcripts are likely to be non-functional but could occasionally activate independent transcription of a poised gene by altering the chromatin environment.

Overall, PCF11-mediated gene punctuation appears essential to promote efficient gene expression of closely spaced active genes. Additionally, it isolates inactive genes from active upstream genes (Figure 4E).

### PCF11 Is Substoichiometric to the CPA Complex

**Figure 5:**
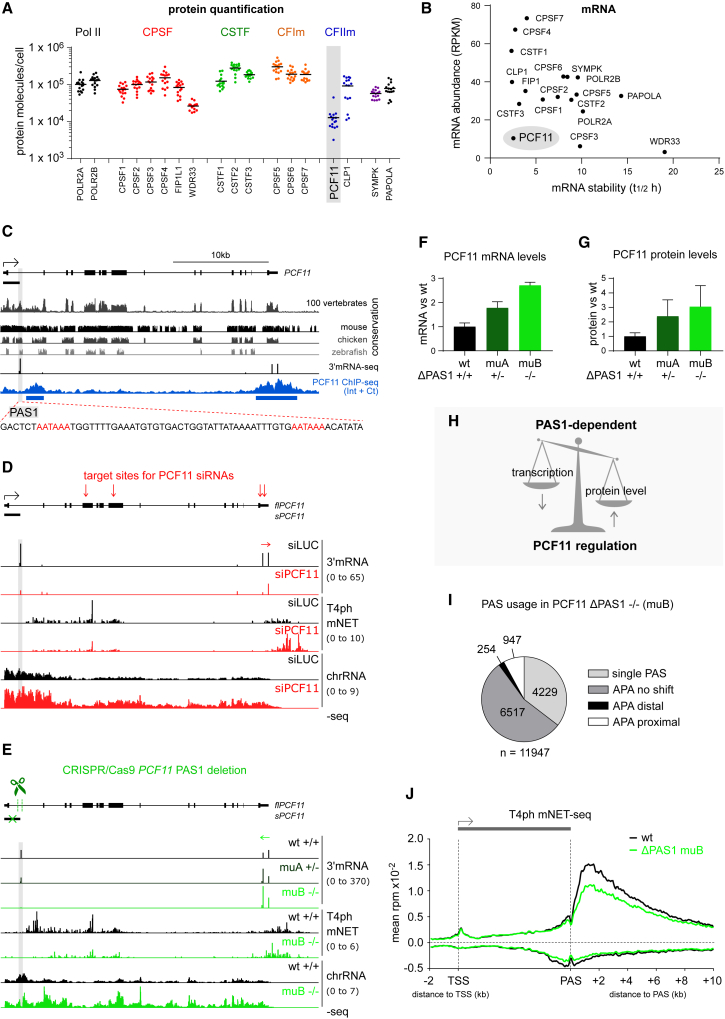
PCF11 Is Substoichiometric to CPA Complex and Is Autoregulated by Premature CPA and Termination (A) Scatterplot of Pol II and CPA subcomplexes protein molecules per cell in colorectal adenoma (Wiśniewski et al., 2015a). n = 16, horizontal lines corresponds to mean. Data from other tissues and cells: Figure S4F and Table S1. (B) Scatterplot of mRNA abundance versus stability of the same factors as (A) in HeLa cells (Tani et al., 2012). (C) Genomic profile of *PCF11* showing evolutionary conservation in 100 vertebrates (top track) and in individual species (middle tracks). Actively used PAS measured by 3′mRNA-seq, PCF11 ChIP-seq signal, and PCF11 enrichment (blue bars) are shown for human cells. Viewing range was auto-scaled to data. Gray shading: conserved PAS1 in first intron. Bottom: DNA sequence, tandem AATAAA hexamers in red. (D and E) Genomic profiles of *PCF11* gene upon PCF11 manipulations. Horizontal arrows show direction of APA. (D) PCF11 depletion. Top: schematic of *PCF11* indicating locations of siRNA target sites (vertical red arrows). Tracks: comparison of 3′mRNA-seq, T4ph mNET-seq, and chrRNA-seq ± PCF11. (E) PAS1 deletion. Top: schematic of *PCF11* indicating a 285 bp CRISPR/Cas9-mediated deletion in the 3 kb first intron, removing PAS1. Tracks are as in (D) for wild-type (wt) cells and CRISPR/Cas9 clones with a partial deletion (muA +/-) and full deletion (muB −/−) of PAS1. Further clones are shown in Figure S5E. (F) Quantification of full-length *PCF11* mRNA levels in wt and PAS1 deletion clones based on 3′mRNA-seq in the *PCF11* 3′ UTR (error bars correspond to SD, n = 3). (G) Quantification of PCF11 WB signal in wt and PAS1 deletion clones. Error bars correspond to SD from two sample dilutions loaded in three WB experiments (n = 6). Representative WB and additional deletion clones are in Figure S5F. (H) Model: PCF11 protein levels modulate transcription of *PCF11* in a PAS1-dependent manner, allowing autoregulation. (I) Pie chart of genome-wide PAS usage in ΔPAS1 clone muB versus wt cells. (J) Meta-gene analysis of T4ph mNET-seq signal in wt cells and ΔPAS1 clone muB (n = 8,389).

The selective association of PCF11 on genes (Figure 4) led us to assess PCF11 protein levels relative to Pol II and other 3′ processing factors, taking advantage of global quantitative proteomics. Notably PCF11 is substoichiometric to other CPA complex subunits in different human cells and tissues: 10- to 20-fold fewer molecules per cell than other CPA and Pol II subunits (Figures 5A and S4F; Table S1). PCF11 is also the least abundant human CID-containing protein (Figure S4G). Consistently, *PCF11* mRNA levels were among the least abundant and most unstable CPA mRNA, as measured by 5′-bromouridine IP chase sequencing (BRIC-seq) (Figure 5B) (Tani et al., 2012). These low PCF11 protein and mRNA levels imply transcriptional regulation. Strikingly, *PCF11* has an evolutionarily conserved first intron, harboring conserved tandem AATAAA poly(A) signals (Figures 5C and S5A) collectively referred to as PAS1. The short *PCF11* isoform resulting from PAS1 usage encodes an ORF with a C-terminal extension. However, this polypeptide was not detected by mass spectrometry analysis in HeLa, U2OS, and HEK293 cell lines.

Interestingly, PCF11 was not only enriched at the 3′ end of its own gene, but also downstream of PAS1 (Figure 5C), suggesting that its low expression could be due to autoregulation by APA and premature termination.

### PCF11 Is Autoregulated by Premature CPA and Termination

Autoregulation of PCF11 by premature termination predicts that downregulation of PCF11 should lead to a decrease in PAS1 usage. We therefore depleted PCF11 by siRNA that specifically targeted full-length *PCF11* RNA (*flPCF11*) but not the short *sPCF11* isoform (Figure 5D). Notably, PAS1 usage and *sPCF11* levels decreased 5-fold upon PCF11 depletion, two times more compared to the directly targeted *flPCF11* (Figures 5D and S5B). Accordingly, T4ph mNET-seq signal downstream of PAS1 decreased and instead increased at the 3′ end of *PCF11*. Also, chrRNA signal increased across the whole gene (Figure 5D). This suggests that PAS1 usage depends on PCF11 levels and that PAS1-linked premature termination regulates *flPCF11* transcription. Interestingly, PAS1 appears particularly sensitive to PCF11 levels, compared to other 3′ processing factors. Re-analysis of data from a published mouse database (Li et al., 2015) revealed that depletion of mCFI-68, mPABPC1, and mPABPN1 increased PAS1 usage in nuclear RNA, whereas mFip1 depletion caused a smaller reduction compared to mPcf11 (Figure S5C).

To directly demonstrate the autoregulatory role of *PCF11* PAS1, we specifically deleted PAS1 including its flanking sequences (285 bp) from the ∼3 kb intron by CRISPR/Cas9 (Figures 5E and S5D). Since we obtained only one full *PCF11ΔPAS1* mutant clone (muB) out of ∼100 single-cell colonies tested, we also included in our analysis three partial deletion mutant clones (muA, muC, and muD; Figure S5D). Consistent with a negative regulatory role of PAS1 on PCF11 expression, all four mutant clones displayed increased *flPCF11* mRNA levels as measured by 3′ mRNA-seq (Figures 5E, 5F, and S5E) and an increase in PCF11 protein levels (Figures 5G and S5F). Additionally, T4ph mNET-seq in muB showed a reduction in intragenic signal with a concomitant 3′ end increase, while chrRNA signal increased across *PCF11* (Figure 5E). In conclusion, PAS1-linked APA and premature termination balances *PCF11* transcription and so maintains stable, low levels of PCF11 protein expression (Figure 5H).

Since the deletion of *PCF11* PAS1 induces PCF11 overexpression, we examined its genome-wide effect on APA and transcription termination. PCF11-overexpressing cells show a preference for proximal APA usage (Figures 5I and S5G) and a smaller window of T4ph mNET-seq signal (Figure 5J), consistent with early CPA and termination caused by the increased levels of PCF11. PCF11-dosage-dependent effects on APA and termination are exemplified by *PCF11* itself: 3′ UTR PAS usage and T4ph mNET-seq signal both shifted distally upon PCF11 reduction (Figure 5D) and proximally upon PCF11 increase (Figure 5E). T4ph mNET-seq signal was specifically downregulated at gene 3′ ends in muB cells (Figure 5J); however, neither PCF11 depletion nor upregulation affected global T4ph levels (Figure S5I). The decrease in T4ph mNET-seq signal may result from more efficient CPA due to high PCF11 levels. PCF11 depletion had a stronger effect than PCF11 upregulation—with more APA distal shifts versus proximal shifts (Figure 2B versus Figure 5I) and strong downregulation of gene expression versus mild upregulation (Figure 4C versus Figure S5H). In conclusion, PCF11 depletion and overexpression show opposite genome-wide effects on APA and termination, implying direct control by PCF11.

### PCF11 Is Essential and Undergoes PAS1-Dependent Autoregulation during Vertebrate Development

**Figure 6:**
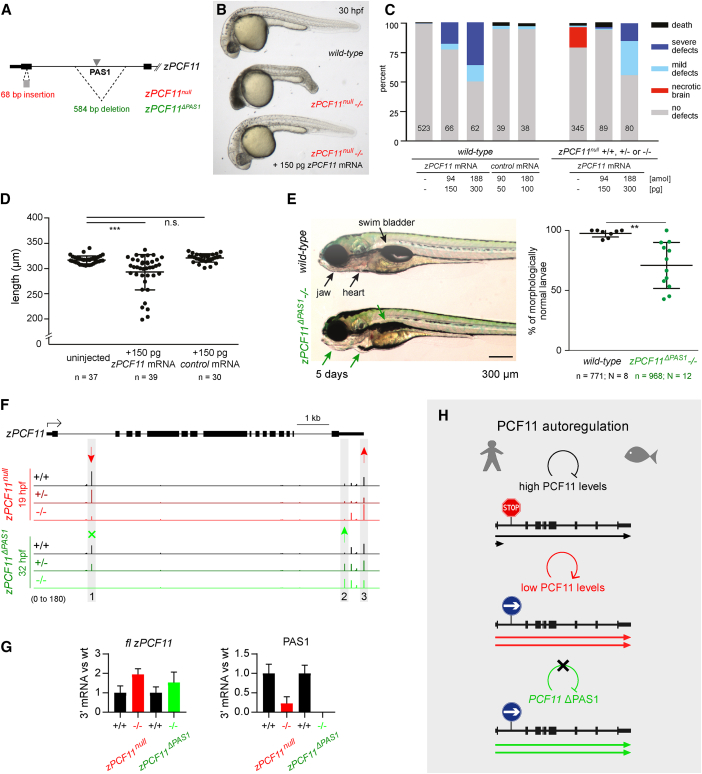
Zebrafish PCF11 Is Essential for Development and Undergoes PAS1-Dependent Autoregulation (A) Schematic of zebrafish *zPCF11*^*null*^ and *zPCF11*^*ΔPAS1*^ mutants. First two exons of *zPCF11* are shown. (B) Severe brain necrosis of *zPCF11*^*null*−/−^ embryos is rescued by injection of 150 pg of *zPCF11* mRNA at the 1-cell stage. (C) Quantification of rescue and overexpression phenotypes upon *zPCF11* mRNA injection. Embryos were scored at 1 day. The numbers within bars indicate number of embryos scored in each treatment group. (C and D) Control mRNA: *GFP-Bouncer* (Herberg et al., 2018). (D) Quantification of decrease in body length at 2 days upon overexpression of *zPCF11* mRNA. Example images of larvae are shown in Figure S6F. (E) Phenotypes observed in *zPCF11*^*ΔPAS1*−/−^ larvae at 5 days. (left) Example images; (right) quantification of morphological defects (lack of swim bladder, heart edema, jaw malformations). n = total number of embryos, N = number of independent crosses. (D and E) Significance determined by unpaired two-tailed t test. (F) 3′ mRNA-seq profiles of the *zPCF11* gene for the indicated genotypes (red: siblings derived from *zPCF11*^*null*+/−^ incrosses; green: siblings derived from *zPCF11*^*ΔPAS1*+/−^ incrosses). Average values of 3–6 biological replicates (individual embryo heads, see Figure S6H). Arrows and gray shading indicate significantly altered PAS usage (DEXseq padj < 0.05). (G) Quantification of *fl zPCF11* mRNA (left) and PAS1 usage (right) in indicated mutants relative to the corresponding wild-type. 3′ mRNA-seq was used for quantification (n > = 3). Error bars correspond to SD. (H) Model of PCF11 autoregulation in human and zebrafish. (top) When PCF11 protein levels are high, *PCF11* transcription is partially attenuated by PAS1 usage and premature termination; as a result, only a fraction of transcripts are full-length. (middle) When PCF11 protein levels are low, PAS1 usage drops leading to more full-length *PCF11* mRNA formation. (bottom) PAS1 removal leads to increased full-length mRNA and protein production.

To obtain more physiological data, we analyzed PAS1 usage in human tissue. Ranking of 22 tissues according to *PCF11* mRNA levels revealed widespread usage of PAS1, with the notable exception of 4 tissues with low *PCF11* expression (Figure S5J). We therefore tested the importance of PCF11 and its PAS1-dependent regulation *in vivo*. We chose zebrafish as model organism since it possesses a conserved (Figure 5C) and active *PCF11* PAS1 (Figure S6A). To assess PCF11 function in vertebrate development, we inactivated zebrafish *pcf11* (*zPCF11*) by generating a CRISPR/Cas9-mediated frameshift mutation (68 bp insertion) in the first coding exon of *zPCF11* (*zPCF11*^*null*^, Figures 6A, S6B, and S6C). While embryos and adult fish heterozygous for the mutation (*zPCF11*^*null*+/−^) were indistinguishable from wild-type (+/+), incrosses of *zPCF11*^*null*+/−^ fish resulted in ∼25% of *zPCF11*^*null*−/−^ embryos with severe brain and CNS necrosis by 20 h post fertilization (hpf) (Figures S6D and 6B), leading to death in 4 days. The fully penetrant brain necrosis could be rescued by *zPCF11* mRNA injection (150 pg) (Figures 6B and 6C), confirming that the defects are due to loss of zPCF11. The initially normal development of *zPCF11*^*null*−/−^ embryos is likely due to the maternal deposition of zPCF11 in the egg (Figure S6E), which supports normal development for the first hours. Together, our analysis provides the first direct evidence that PCF11 is essential for vertebrate development.

Interestingly, our mRNA rescue experiments revealed that injection of higher amounts of *zPCF11* mRNA (300 pg) into embryos derived from *zPCF11*^*null*+/−^ incrosses or overexpression of zPCF11 in wild-type embryos caused a range of morphological abnormalities, including an overall shortening of the body axis (Figures 6C, 6D, and S6F). Thus, both too much and too little zPCF11 interfere with normal development. The necessity of tightly controlled zPCF11 levels *in vivo* prompted us to investigate the importance of the conserved intronic PAS1 in balancing zPCF11 during zebrafish development. We therefore generated zebrafish mutants with the conserved intronic PAS1 deleted by CRISPR/Cas9 (*zPCF11*^*ΔPAS1*^) (Figures 6A, S6B, and S6C). Homozygous mutant *zPCF11*^*ΔPAS1*−/−^ larvae showed reduced fitness such as delayed swim bladder formation, mild jaw abnormalities, and weak edema formation at 5 days (Figure 6E). Although most *zPCF11*^*ΔPAS1*−/−^ larvae developed into phenotypically normal adults, the presence of a larval phenotype in the absence of PAS1 suggests PCF11 autoregulation. Consistently, *zPCF11*^*ΔPAS1*−/−^ embryos showed increased zPCF11 protein levels when compared to wild-type embryos (Figure S6G).

To confirm that PAS1-mediated PCF11 autoregulation occurs during zebrafish development, 3′ mRNA-seq was performed on *zPCF11*^*null*^ and *zPCF11*^*ΔPAS1*^ mutant versus wild-type embryos. Heads of 3–6 single embryos were sequenced individually for each genotype (+/+, +/−, −/−). 3′ mRNA-seq of *zPCF11*^*null*−/−^ embryos consistently showed that lack of zPCF11 protein leads to 4- to 5-fold lower PAS1 usage, and a concomitant 2-fold increase in *fl zPCF11* mRNA (Figures 6F, 6G, and S6H). In contrast, and consistent with our immunostainings (Figure S6G), *zPCF11*^*ΔPAS1*−/−^ embryos showed an about 1.5-fold increase in *fl zPCF11* mRNA levels (Figures 6F, 6G, and S6H).

Globally, *zPCF11*^*null*^ and *zPCF11*^*ΔPAS1*^ mutant zebrafish embryos revealed few statistically significantly changes in APA (Figure S6I). This may be partly because zebrafish embryos comprise a heterogenous cell population; analysis of average PAS usage could therefore result in high variability of detected PAS usage between the individual wild-type embryos (Figure S6H). Nevertheless, we observed a general tendency for more distal APA in *zPCF11*^*null*−/−^ mutants, and proximal APA in *zPCF11*^*ΔPAS1*−/−^ mutants (Figure S6I), matching our findings in human cells (Figures 2B and 5I). *zPCF11* appears to be an APA-prone gene during zebrafish embryogenesis (Figure 6F) as there was a significant distal APA shift in the *zPCF11* 3′ UTR in *zPCF11*^*nul*−/−^ embryos, and a proximal shift in *zPCF11*^*ΔPAS1*−/−^ embryos. These data together suggest that zPCF11 favors proximal PAS usage, like its human homolog.

We conclude that, both in human cell lines and during zebrafish development, PAS1-linked premature termination promotes PCF11 autoregulation and homeostasis (Figure 6H), which increases animal fitness.

### Transcriptional Regulators Are Controlled by PCF11-Dependent Premature CPA and Termination

**Figure 7:**
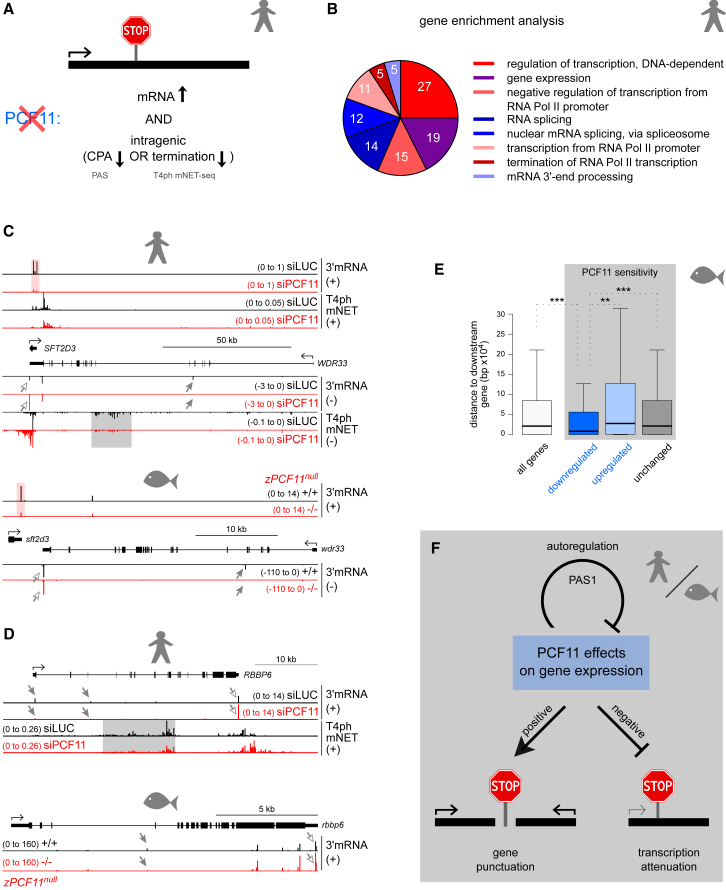
Transcriptional Regulators Are Controlled by PCF11-Dependent Premature CPA and Termination (A) Criteria for identifying genes attenuated by PCF11-dependent premature CPA/termination: these are significantly upregulated upon PCF11 depletion (DEseq padj < 0.05) and either show a >2-fold decreased intragenic T4ph mNET-seq signal or possess a significantly decreased PAS (DEXseq padj < 0.1). (B) Enrichment analysis of GO biological process for transcripts attenuated by PCF11 in human cells. Numbers in pie chart correspond to number of genes in each category. GeneCodis3 software was used (padj < 0.01 and gene number >2). Red shades, genes related to transcription; blue shades, genes related to RNA processing. (C and D) Genomic profiles ± PCF11 of *WDR33* (C) and *RBBBP6* (D) for human cells (top) and zebrafish embryos (*zPCF11*^*null*^, bottom). Gray shading highlights decreased intragenic T4ph mNET-seq signal, and arrows highlights distal APA in PCF11-depleted conditions (gray arrowheads: decreased intragenic PAS signal, white arrowheads: increased 3′ UTR PAS usage). (E) Gene distance analysis for genes significantly downregulated, upregulated or unchanged in *zPCF11*^*null*−/−^ versus ^+/+^ embryos. Statistical significance was tested by Mann-Whitney test. (F) Model: PCF11 displays opposing functions in gene expression. PCF11 punctuates closely spaced genes, leading to their gene expression enhancement. In contrast, PCF11 negatively affects the expression of a subset of transcriptional regulators by attenuating their transcription. PCF11 is also autoregulated by PAS1-dependent premature CPA and termination.

We demonstrate above that PCF11 regulates its own expression by premature termination and favors proximal PAS usage and early termination genome-wide. This underlines the possibility that PCF11 attenuates the transcription of other genes. We therefore searched our human datasets for pc genes significantly upregulated upon PCF11 depletion that also show decreased intragenic termination (T4ph mNET-seq signal) or CPA (PAS usage, Figure 7A). 218 genes were identified as candidates for hPCF11-induced attenuation (Table S2). 55 genes showed simultaneously decreased CPA and termination (Figure S7A). Premature termination in the absence of a detectable polyadenylated product most likely reflects the unstable nature of such transcripts (Chiu et al., 2018). Oppositely, decreased intragenic PAS usage without associated changes in termination may reflect uncoupling of CPA and termination, as described in Figure 2. Strikingly, gene ontology (GO)-term analysis of genes attenuated by PCF11 revealed that they are highly enriched for regulators of gene expression, both at the level of transcription and RNA processing (Figure 7B), even for the CPA and termination criteria applied independently (Figures S7C and S7D). We then repeated the CPA-based analysis for the zebrafish dataset and identified 108 putative zPCF11-attenuated genes, again enriched for genes involved in transcription (Figure S7E). Notably, PCF11-dependent attenuation can be observed on the same genes in human and zebrafish, especially 3′ mRNA processing factors (Figures 7C, 7D, and S7B). Thus, both in human cells and during zebrafish development, PCF11 downregulates a subset of transcriptional regulators by premature CPA and termination.

Finally, we determined whether PCF11 positively affects closely spaced genes in zebrafish, as in human. Indeed, gene distance analysis revealed that zPCF11-sensitive downregulated genes have a significantly closer downstream neighbor, compared to both zPCF11-insensitive and upregulated genes (Figure 7E; note also the downregulation of human and zebrafish *SFT2D3* in Figure 7C).

We conclude that the selective functions of PCF11 in punctuating closely spaced genes and attenuating transcription of gene expression regulators are conserved in vertebrates from zebrafish to human (Figure 7F).

## Discussion

### The Role of PCF11 in PAS Selection and Transcriptional Termination

We present a systematic study of PCF11 in vertebrate gene expression. PCF11 is indispensable for zebrafish development, while in human cells it is required for efficient CPA and transcription termination genome-wide. Furthermore, PCF11 levels in human cells and zebrafish embryos determine alternative polyadenylation patterns: decreased PCF11 levels result in globally more distal PAS usage, whereas increased PCF11 levels (as a result of PAS1 deletion) induced proximal PAS usage. This is in agreement with a previously published mouse screen (Li et al., 2015). We observed fewer significant APA events in zebrafish embryos compared to human cell culture. This may be due to technical reasons (see above), or alternatively APA regulation may reflect an enhanced role for PCF11 in evolution. Notably in *S. pombe*, Seb1, but not Pcf11, affected alternative 3′ ends on selected transcripts (Larochelle et al., 2017).

PCF11 protein levels are substoichiometric in multiple human tissues and cell lines: an order of magnitude lower than the average number of molecules per cell of other CPA complex subunits. This suggests that human PCF11 is an accessory or regulatory factor rather than a core subunit of the CPA complex. Consistently, a previous biochemical study reported that CFIIm (containing PCF11) associates only weakly or transiently with the CPA complex (Shi et al., 2009). We suggest that PCF11 acts selectively. Thus, PCF11 ChIP-seq analysis shows binding to a large proportion of transcription units—including transcript classes that can be processed independently of the CPA machinery. At the same time, PCF11 binding was undetectable on some active genes undergoing polyadenylation. Since we employed two independent polyclonal antibodies, it is unlikely that our results are due to epitope inaccessibility. However, it is possible that antibody sensitivity, fast RNA processing, or transient PCF11 binding preclude PCF11 ChIP detection on some genes. In addition to a selective PCF11 ChIP profile, we found that transcription termination defects and APA changes upon PCF11 depletion were widespread, but not universal. Two previous findings support the view that PCF11 is a selective factor in metazoans. First, staining of *Drosophila* polytene chromosomes with PCF11 antibody correlated with Pol II staining, but fewer bands were visible (Zhang and Gilmour, 2006). Second, on the HIV provirus, PCF11 was required for 5′ long terminal repeat (LTR) but not 3′ LTR termination (Zhang et al., 2007). Therefore, PCF11 may act as a selective 3′ processing and termination factor in metazoans.

If PCF11 affects only a subset of genes, alternative factors may exist to substitute its function. Possible candidates are the more abundant but uncharacterized mammalian CID containing proteins SCAF4/8, RPRD1A/B, and/or RPRD2. In *S. pombe*, both PCF11 and the SCAF4/8 homolog Seb1 independently contribute to CPA and transcription termination (Lemay et al., 2016, Wittmann et al., 2017).

### Regulation of PCF11 Expression

*PCF11* contains a strong and evolutionary conserved PAS within its first intron (PAS1). PAS1 enables autoregulation by premature CPA and termination, which we demonstrate here in human cell culture and during zebrafish development. Independently of our study, the Tian laboratory has found PAS1-mediated PCF11 autoregulation in murine cells (Wang et al., 2019). We note that PAS1 renders *PCF11* expression sensitive to transcription and RNA processing dynamics. Accordingly, slow Pol II elongation, splicing inhibition, or UV treatment lead to almost exclusive transcription of the *sPCF11* isoform, attenuating *flPCF11* expression (Figure S7F). *PCF11* mRNA levels fluctuate widely in control conditions (Figure S5B). Due to both its low levels and stability, transcriptional changes of *PCF11* may have relatively fast functional effects. We speculate that in conditions where elongation or RNA processing are suboptimal (e.g., after UV damage) it is beneficial to downregulate PCF11 and so reduce CPA and termination efficiency. This may act to counteract the global shortening of transcripts that occurs under such conditions (Devany et al., 2016, Williamson et al., 2017).

### Premature Termination as a Regulatory Paradigm in Vertebrates

PCF11 activity displays opposing functions in gene expression. It positively affects expression levels of many genes—especially closely spaced ones, which cannot employ failsafe termination mechanisms. Interestingly, such genes are also prone to defective termination under cellular stress conditions (Vilborg et al., 2017). In contrast, we find that unperturbed cells downregulate a subset of genes by PCF11-mediated transcriptional attenuation, and this function is conserved in vertebrates from zebrafish to human. The phenomenon of negative gene regulation by premature termination is well described in *S. cerevisiae*, where it plays a physiological role in response to changing growth conditions (Colin et al., 2011). In the mammalian system, genome-wide premature termination of pc gene transcription has been previously reported in cells depleted for U1 snRNP (Kaida et al., 2010) and upon DNA damage (Devany et al., 2016, Williamson et al., 2017). Interestingly, Pol II accumulation at human and *Drosophila* promoters is not solely due to Pol II pausing but also associated with premature termination (Nojima et al., 2015, Krebs et al., 2017). We predict that premature termination is a widespread gene regulatory mechanism in metazoans. Premature termination of pc genes in budding yeast is mediated by the Nrd1-Nab3-Sen1 (NNS) complex, which additionally auto-regulates Nrd1 levels by attenuating NRD1 transcription when its levels are high (Arigo et al., 2006). Given the lack of a direct homolog for the NNS complex in higher eukaryotes, we speculate that in vertebrates PCF11 might be a functional counterpart of yeast Nrd1. Interestingly, yeast PCF11 gene is also controlled by NNS-dependent premature termination (Creamer et al., 2011) and cooperates with the NNS complex (Grzechnik et al., 2015). Overall, we predict that premature termination is a fundamental gene regulatory mechanism conserved in all eukaryotes.

## STAR★Methods

### Key Resources Table

**Table:** 

REAGENT or RESOURCE	SOURCE	IDENTIFIER
**Antibodies**		

Rabbit polyclonal anti-Pol II total (N-20) (used for: ChIP-seq)	Santa Cruz	Cat# sc-899; RRID: AB_632359
Mouse monoclonal anti-Pol II CTD S2ph (used for: WB, ChIP-seq)	MBL international	Cat# MABI0602; RRID: AB_2747403
Rat monoclonal anti-Pol II CTD T4ph (clone 6D7) (used for: mNET-seq)	Active motif	Cat# 61361; RRID: AB_2750848
Rabbit polyclonal anti-Pol II CTD T4ph (used for: WB)	Novus biologicals	Cat# NBP1-49546; RRID: AB_10011602
Rabbit polyclonal anti-PCF11 (PCF11-Ct) (used for: WB, IP, ChIP-seq)	Abcam	Cat# ab134391; RRID: AB_2783786
Rabbit polyclonal anti-PCF11 (PCF11-Int) (used for: WB, IP, ChIP-seq, IF)	Bethyl	Cat# A303-705A; RRID: AB_11205447
Rabbit polyclonal anti-CPSF73 (used for: ChIP-seq)	Bethyl	Cat# A301-091A; RRID: AB_2084528
Rabbit polyclonal anti-H2Av (used for: ChIP-seq spike-in)	Active motif	Cat# 61686; RRID: AB_2737370
Mouse monoclonal anti-CDH1 (used for: IF)	BD Biosciences	Cat# 610181; RRID: AB_397580
Goat polyclonal anti-rabbit (used for: WB)	Li-COR	Cat# 926-32211; RRID: AB_621843
Goat polyclonal anti-mouse (used for: WB)	Li-COR	Cat# 926-68070; RRID: AB_10956588
Goat polyclonal anti-rabbit AlexaFluor-488 (used for: IF)	Thermo Fisher Scientific	Cat# A-11034; RRID: AB_2576217
Goat polyclonal anti-mouse AlexaFluor-546 (used for: IF)	Thermo Fisher Scientific	Cat# A-11003; RRID: AB_2534071

**Chemicals, Peptides, and Recombinant Proteins**		

Puromycin dihydrochloride	Sigma-Aldrich	Cat# P8833
NuPAGE 3-8% Tris-Acetate Protein Gel, 10 well	ThermoFisher Scientific	Cat# EA0375BOX
Novex 6% TBE gel, 12 well	ThermoFisher Scientific	Cat# EC62652BOX
Novex 6% TBE-Urea (TBU) gel, 12 well	ThermoFisher Scientific	Cat# EC68652BOX
TURBO DNase	ThermoFisher Scientific	Cat# AM2238
T4 polynucleotide kinase (PNK), 3′ phosphatase minus	NEB	Cat# M0236S
T4 RNA ligase, deletion mutant 2	Epicenter	Cat# LR2D1132K

**Critical Commercial Assays**		

MicroPlex library preparation kit v2	Diagenode	Cat# C05010012
TruSeq small RNA library preparation kit	Illumina	Cat# RS-200-0012
NEBNext Ultra II Directional RNA Library Prep Kit for Illumina	NEB	Cat# E7760
NextSeq High-Output v2 Kit, 75 cycles	Illumina	Cat# FC-404-2005
Superscript III first strand synthesis system	Thermo Fisher	Cat# 18080051
Ribo-Zero Gold rRNA removal kit (H/M/R)	Illumina	Cat# MRZG12324
QuantSeq 3′mRNA-Seq library prep kit REV for Illumina	LEXOGEN	Cat# SKU 016.24
QuantSeq 3′mRNA-seq Library Prep Kit FW for Illumina	LEXOGEN	Cat# SKU 015.96

**Deposited Data**		

Raw and processed NGS data (mNET-seq, 3′ mRNA-seq, chromatin RNA-seq and ChIP-seq)	This paper	GEO: GSE123105
Raw image files	This paper; Mendeley Data	https://doi.org/10.17632/rmjm32hd6n.1
Re-analyzed global quantitative proteomics data	Wiśniewski lab	Nagaraj et al., 2011, Wiśniewski et al., 2015b, Wiśniewski et al., 2015a, Wiśniewski et al., 2016 (supplemental table in the relevant publication)
Re-analyzed mRNA stability and abundance data	Tani et al., 2012	https://genome.cshlp.org/content/suppl/2012/02/14/gr.130559.111.DC1/Tani_Supp_Tables_revised2.xls
Re-analyzed 3′READS data	Li et al., 2015	GEO: GSE62001
Re-analyzed SAPAS data from human tissues	You et al., 2015	http://genome.bucm.edu.cn/utr/
Re-analyzed zebrafish RNA-seq and 3P-seq data	Ulitsky et al., 2011, Pauli et al., 2012, Herberg et al., 2018	GEO: GSE32880, GSE32900, GSE111882
Re-analyzed Pol II S2ph ChIP-seq data from fast, normal and slow Pol II mutant cell lines	Fong et al., 2017	GEO: GSE97827
Re-analyzed nucleoplasmic RNA-seq data	Nojima et al., 2015	GEO: GSE60358

**Experimental Models: Cell Lines**		

HeLa (human)	Proudfoot lab	N/A
HeLa Flp-In TRex (human) established by Elena Dobrikova and Matthias Gromeier, Duke University Medical Center	Gromaier lab	N/A

**Experimental Models: Organisms/Strains**		

*Danio rerio* (zebrafish)	Pauli lab	N/A

**Oligonucleotides**		

siLUC (custom siRNA)	Sigma-Aldrich	Sequence (5′-3′)
		Sense: GAUUAUGUCCGGUUAUGUAUU
		Antisense: [phos]UACAUAACCGGACA UAAUCUU
siPCF11 (human) ON-TARGETplus SMARTpool	Dharmacon	L-015381-01
See Table S3 for primers used in this study.		

**Recombinant DNA**		

Plasmid: epX459(1.1) (a modified version pX459 V2.0 Addgene plasmid # 62988 wherein WT SpCas9 is replaced with engineered eSpCas9(1.1))	Joey Riepsaame	N/A
Plasmid: CRISPR_V076	This paper	N/A
Plasmid: CRISPR_V078	This paper	N/A

**Software and Algorithms**		

GENCODE release 19		https://www.gencodegenes.org/
FastQC		http://www.bioinformatics.babraham.ac.uk/projects/fastqc/
Bowtie2 (version 2.3.2)		http://bowtie-bio.sourceforge.net/bowtie2/index.shtml
MACS2 (version 2.1.1.20160309)		https://github.com/taoliu/MACS
STAR (version 2.5.2b)		https://github.com/alexdobin/STAR
Bioconductor (version 3.7)		https://www.bioconductor.org/
Cutadapt		https://cutadapt.readthedocs.io/en/stable/installation.html
bam2fastx (TopHat 2 component)		http://ccb.jhu.edu/software/tophat/index.shtml
BBtools		https://sourceforge.net/projects/bbmap/
DEXseq (version 1.28.0)		https://bioconductor.org/packages/DEXSeq
DEseq2 (version 1.18.1)		https://doi.org/10.18129/B9.bioc.DESeq2
Ggplot2 (version 3.0.0)		https://cran.r-project.org/web/packages/ggplot2/index.html
GeneCodis3		http://genecodis.cnb.csic.es/
All above software and packages were used on linux (ubuntu 16.04) mostly within the R (3.4.3) environment.		

### Contact for Reagent and Resource Sharing

Further information and requests for resources and reagents should be directed to the lead contact, Nick J. Proudfoot (nicholas.proudfoot@path.ox.ac.uk).

### Experimental Model and Subject Details

#### Cell lines

All human cell culture experiments were performed in HeLa cells, either wild-type or engineered HeLa Flp-In TRex (established by Elena Dobrikova and Matthias Gromeier, Duke University Medical Center). Cells were cultivated at 37°C and 95% humidity with 5% CO2 in Dulbecco’s Modified Eagle’s Medium (DMEM), high glucose (4,5 g/l) with 10% fetal calf serum (FCS, Perbio) and 1% L-Glutamine (200 mM).

#### Zebrafish

Zebrafish (*Danio rerio*) were raised according to standard protocols (28°C water temperature, 14/10 hr light/dark cycle). TLAB fish, generated by crossing zebrafish AB and the natural variant TL (Tupfel Long-fin) stocks, served as wild-type zebrafish for all experiments. *zPCF11*^*null*^ and *zPCF11*^Δ*PAS1*^ mutant zebrafish were generated as part of this study and are described in detail below. All fish experiments were conducted according to Austrian and European guidelines for animal research and overseen by an institutional animal committee. All fish experiments were approved by local Austrian authorities (animal protocol GZ: 342445/2016/12).

### Method Details

#### siRNA transfection

siRNA treatment was performed using Lipofectamine RNAimax (Thermo) as described in the product manual. A pool of 4 siRNAs was used to target PCF11 (Dharmacon ON-TARGETplus SMARTpool L-015381-01) as well as a control siRNA against Luciferase (siLUC, see Key Resources Table). The efficiency of depletion was tested by western blot. Initial 24-72 hr time course and a 2-50 nM concentration range test was performed to determine optimal knock-down conditions (Figures S1A and S1B). All genomic knock-down experiments were performed for 48 hr. To avoid indirect effects due to decreased Pol II S2 phosphorylation at high siPCF11 concentrations (Figure S1B), 5 nM siRNA concentration was chosen as the standard condition. Some replicate genomics experiments were additionally performed using 50nM siRNA, as indicated in the genomic dataset description in GEO. The 5 nM and 50 nM treatments gave similar genome-wide effects therefore had been treated as biological replicates.

#### Immunoblotting

Proteins were resolved by electrophoresis using 3%–8% Tris-Acetate gels (NuPAGE) that separate the migration of PCF11 and Pol II proteins, and blotted onto nitrocellulose membranes. Blots were probed with the antibodies described Key Resources Table, and visualized on a Li-COR Odyssey machine. Li-COR software was used for quantifications.

#### Deletion of PCF11 PAS1 in human cells by CRISPR/Cas9

Protospacer sequences were cloned into BbsI sites of column-purified plasmid epX459(1.1), a modified version pX459 V2.0 (gift from Feng Zhang (Addgene plasmid # 62988)) wherein WT SpCas9 is replaced with engineered eSpCas9(1.1) (gift from Feng Zhang (Addgene plasmid # 71814)) via KflI/ApaI subcloning. Briefly, equimolar amounts (10 uM; 10 ul) of overlapping oligos harboring the appropriate sgRNA target sequences were phosphorylated (T4 PNK, NEB) and annealed for 5 min. at 95° before slowly cooling to room temperature. Phosphorylated and annealed oligos were subsequently ligated (T4 ligase, NEB) overnight at room temperature into BbsI-digested epX459(1.1) (5:1 insert-to-plasmid ratio). Upon E.coli (DH10b) transformation and ampicillin selection, plasmid DNA of individual inoculated bacterial clones was prepped (QIAprep Spin Miniprep kit, QIAGEN) and correctly cloned protospacer sequences verified using Sanger sequencing (using the primer tandem_sgRNAs_seq - TTCGCCACCTCTGACTTGAGCGT). The following oligos were used for protospacer cloning: PCF11-PAS1_1_F (5′- caccGACCGTCTCTAAACAATATAT −3′) and R (5′- aaacATATATTGTTTAGAGACGGTC-3′); PCF11-PAS1_2_F (5′- caccGACAAGATACACGGTTTCAGG-3′) and R (5′- aaacCCTGAAACCGTGTATCTTGTC-3′). Guide RNA/Cas9 expression vectors were transfected into HeLa Flp-In TRex cells using Lipofactamine 2000 (Thermo Fisher Scientific) according to manufacturer’s instructions. 24 hr after transfection puromycin was added to the cells at 3 μg/ml concentration to select for plasmid-expressing cells. After 24 hr of puromycin selection, the medium was exchanged for non-selective conditions and cells were left to recover for 72 hr before sorting single cells by FACS into four 96 well plates. Individual clones were screened for PAS1 deletion using PCR and the nature of the deletion of candidate clones was verified by Sanger sequencing using primers PCF11_PAS1_genotyping_F and R, shown in Table S3. Initially obtained clones were wild-type, PCF11 PAS1 deletion clones only generated colonies 1-2 weeks after normal clones, indicating possible early cell cycle block in the mutant cells.

#### ChIP-sequencing

Cells were cultivated on 150 mm dishes until 70% confluency, fixed by addition of 1% formaldehyde for 15 min at 37°C and quenched by addition of glycine (125mM) for 5 min. The cells were collected by scraping on ice, washed 3 times with cold PBS, resuspended in 1.5 mL L1 buffer (50 mM Tris pH 8.0; 2 mM EDTA pH 8.0; 0,1% NP40; 10% glycerol; protease inhibitors) per 10^7^ cells, and lysed on ice for 5 min. The nuclei were collected by centrifugation at 800 g for 5 min at 4°C and lysed in 1,5 mL of L2 buffer (0,2% SDS; 10 mM EDTA; 50 mM Tris pH 8.0; protease inhibitors). The suspension was sonicated in 15 mL conical tubes in a cooled Bioruptor (Diagenode) for 15 min at high settings, and cleared by centrifugation for 10 min at 13000 rpm. The chromatin (DNA) concentration was quantified using NanoDrop (Thermo Scientific) and the sonication efficiency monitored on an agarose gel. Protein A and protein G dynabeads (Thermo Fisher Scientific, combined 1:1) were blocked with BSA (250mg/ml beads) in dilution buffer (0,5% NP40; 200 mM NaCl; 50 mM Tris pH 8.0; protease inhibitors) for 2 hr in cold room. The chromatin was diluted 10x in the dilution buffer. For calibration of selected samples (indicated in GEO record), 25 ng of *Drosophila* chromatin was added per 100 μg of human chromatin (Egan et al., 2016). The chromatin was pre-cleared with blocked beads for 1 hr at 4°C. 100 μg of pre-cleared chromatin was incubated with 10 μg of α-PCF11 or α-Pol II antibody and 0.5 μg of *Drosophila*-specific α-H2Av O/N at 4°C, then with 60 μL blocked beads for further 1-2 hr at 4°C. The beads were washed 2x with WB-150 (0.02% SDS; 0.5% NP40; 2 mM EDTA; 150 mM NaCl; 20 mM Tris pH 8.0), 3x with WB-250 (0.02% SDS; 0.5% NP40; 2 mM EDTA; 250 mM NaCl; 20 mM Tris pH 8.0), 2x with WB-500 (0.02% SDS; 0.5% NP40; 2 mM EDTA; 500 mM NaCl; 20 mM Tris pH 8.0) and finally 1x again with WB-150. The immuno-complexes were eluted by two 15 min incubations at 30°C with 100ul elution buffer (1% SDS, 100mM NaHCO3), and de-crosslinked for 4 hr at 65°C in the presence of 10U RNase A. The immunoprecipitated DNA was then purified with the MinElute PCR purification kit (QIAGEN) according to manufacturer’s protocol and used for library preparation. For PCF11 and CPSF73 ChIP samples, Diagenode MicroPlex library preparation kit v2 (C05010012) was used to prepare libraries for sequencing, following manufacturer’s instructions. Indexed libraries were quantified, normalized and pooled for sequencing on Illumina NextSeq550 system. Pol II ChIP experiments were performed similarly, however the genomic libraries were prepared using NEBNext ChIP-Seq master-mix kit and sequenced on a 50-bp single-end run using the Illumina HiSeq 2000 platform. Two biological replicates of Pol II total and CPSF73 ChIP experiments were performed in siLUC and siPCF11 conditions. PCF11 ChIP experiments were performed using two independent antibodies, each in two biological replicates.

#### Mammalian Native Elongating Transcript sequencing (mNET-seq)

Detailed protocols for mNET-seq were previously described (Nojima et al., 2015, Nojima et al., 2016). In brief, the chromatin fraction was isolated from 3x10^7^ HeLa cells. Chromatin was digested in 100 μL of MNase (40 units/ μL) reaction buffer for 5-18 min at 37°C in a thermomixer (1,400 rpm). After addition of 10 μL EGTA (25mM) to inactivate MNase, soluble digested chromatin was collected by 13,000 rpm centrifuge for 5 min. The supernatant was diluted with 400 μL of NET-2 buffer (50 mM Tris-HCl pH 7.4, 150 mM NaCl and 0.05% NP-40) and Pol II antibody-conjugated beads were added. 40 μg of T4ph Pol II antibody was used per sample. Immunoprecipitation was performed at 4°C for 1 hr. The beads were washed with 1 mL of NET-2 buffer six times with 100 μL of 1xPNKT (1xPNK buffer and 0.05% Triton X-100) buffer once in cold room. Washed beads were incubated in 50 μL PNK reaction mix (1xPNKT, 1 mM ATP and 0.05 U/ml T4 PNK 3′phosphatase minus (NEB) in Thermomixer (1,400 rpm) at 37°C for 6 min. After the reaction beads were washed with 1 mL of NET-2 buffer once and RNA was extracted with Trizol reagent. RNA was suspended in urea Dye (7M Urea, 1xTBE, 0.1% BPB and 0.1% XC) and resolved on 6% TBU gel (Invitrogen) at 200 V for 5 min. In order to size select 30-160 nt RNAs, a gel fragment was cut between BPB and XC dye markers. 0.5 mL tube was prepared with 3-4 small holes made with 25G needle and placed in a 1.5 mL tube. Gel fragments were placed in the layered tube and broken down by centrifugation at 12,000 rpm for 1 min. The small RNAs were eluted from gel using RNA elution buffer (1 M NaOAc and 1 mM EDTA) at 25°C for 1 hr in Thermomixer (900 rpm). Eluted RNA was purified with SpinX column (Coster) with 2 glass filters (Millipore) and the flow-through RNA was ethanol precipitated. mNET-seq libraries were prepared using TruSeq small RNA library preparation kit (Illumina, cat. no. RS-200-0012) and user supplied T4 RNA ligase, deletion mutant 2 (Epicenter, cat. no. LR2D1132K), according to Illumina instructions. 13-15 cycles of PCR were used to amplify the library. Before sequencing, the libraries were size-selected on a 6% TBE gel selecting only the 150-230 bp PCR product to exclude primer-primer ligated DNA. Gel elution was performed as described above. The libraries were sequenced on NextSeq500 using NextSeq High-Output Kit, 75 cycles (Illumina). mNET-seq experiments were performed and sequenced as independent biological repeats: 3 repeats of siLUC and siPCF11 experiments, and 2 repeats for wt and muB PCF11ΔPAS1 cells.

#### Chromatin-bound RNA sequencing (chrRNA-seq)

Chromatin-bound RNA-seq protocol was previously described (Nojima et al., 2015). 1x10^7^ cells for each condition were resuspended in 12ml of ice cold PBS. Cells were spun down at 500 g, 5 min at 4°C and cell pellets were resuspended in 800 μL of HLBN hypotonic buffer (10 mM Tris-HCl pH 7.5, 10 mM NaCl, 2.5 mM MgCl_2_, 0.05% NP40). 480 μl of buffer HLBNS (HLBN, 25% sucrose) was carefully under-layered to create sucrose cushion, and nuclei were isolated by centrifugation for 5 min at 1000 g at 4°C. Supernatant containing cytoplasmic debris was discarded and the nuclear pellet was re-suspended in 100 μl of ice-cold buffer NUN1 (20 mM Tris-HCl pH 7.9, 75 mM NaCl, 0.5 mM EDTA, 50% glycerol; 1 mM DTT and cOmplete EDTA free protease inhibitors (Sigma) added fresh). Nuclei were lysed in 1200 μl of ice-cold lysis buffer NUN2 (20 mM HEPES pH7.6, 300 mM NaCl, 7.5 mM MgCl_2_, 0.2 mM EDTA, 1 M urea, 1% NP40; 1 mM DTT) during 15min incubation on ice and RNA-bound chromatin was pelleted at 16000 g for 10min at 4°C. Chromatin-RNA pellet was re-suspended in 200 μl of high salt buffer HSB (10 mM Tris-HCl pH 7.5, 500 mM NaCl, 10 mM MgCl_2_). DNA and proteins were digested with Turbo DNase (Life Sciences) and proteinase K (10 mg/ml, ThermoFisher, nuclease free), incubating on ThermoMixer at 37°C for 10 min and 30min, respectively. RNA was extracted with 1 mL of TRI Regent (Sigma) according to the manufacturer guidelines. RNA was dissolved in 1xTURBO DNase buffer, digested with TURBO DNase for 30 min at 37°C on a ThermoMixer and extracted with TRI reagent. RNA was washed three times with 75% ethanol, and dissolved in water. The RNA integrity was checked on the Agilent 4200 TapeStation system (Agilent Technologies). 1 μg of input RNA was depleted of ribosomal RNA with Ribo-Zero Gold Kit (MRZG12324, Illumina) according to manufacturer’s guidelines. 5 μL of ribo-depleted RNA (i.e., 12-60 ng RNA according to Qubit quantification) was used as input for library preparation. Chromatin RNA-seq libraries from 2-4 biological repeats were prepared with NEBNext Ultra II Directional RNA Library Prep Kit for Illumina (E7760). Libraries were sequenced on NextSeq500 using NextSeq High-Output Kit, 75 cycles (Illumina). ChrRNA-seq experiments were performed and sequenced as independent biological repeats: 2 repeats of siLUC and siPCF11 experiments, and 4 repeats for wt and muB PCF11ΔPAS1 cells.

#### 3′ mRNA-seq on HeLa cells

PAS mapping (3′ mRNA-seq) was performed on nuclear RNA to enrich for newly transcribed RNAs. To this end, cells on 150 mm dishes were grown until 70% confluent and harvested. After centrifugation, the cell pellet was resuspended in 4 mL of ice-cold HLB+N buffer (10 mM Tris pH 7.5, 10 mM NaCl, 2.5 mM MgCl2, 0.5% NP40) and incubated on ice for 5 min. The suspension was then underlayed with 1 mL of ice-cold HLB+NS buffer (10 mM Tris pH 7.5, 10 mM NaCl, 2.5 mM MgCl2, 0.5% NP40, 10% sucrose) and centrifuged at 420 g for 5 min at 4°C. The supernatant was discarded, and the nuclear pellet washed with PBS. RNA was purified from the nuclei using TRI reagent (Sigma) according to manufacturer’s instructions. Residual DNA was digested using 4U Turbo DNase (Life Tech) for 10 min at 37°C followed by proteinase K digestion for 10 min at 37°C. TRI reagent purification and DNase digestion were repeated. RNA was further acid phenol/chloroform and chloroform extracted, followed by ethanol precipitation. The purified RNA was then resuspended in 20ul ultrapure water. 3′ mRNA-seq libraries were prepared using Lexogen QuantSeq 3′ mRNA-Seq Library Prep Kit REV for Illumina according to manufacturer’s instructions, and sequenced on HiSeq2500. 3′ mRNA-seq experiments were performed and sequenced as independent biological repeats: 4 repeats of siLUC and siPCF11 experiments, and 3 repeats for wt, muA and muB PCF11ΔPAS1 cells. We detected a trace amount of RNA from wt cells in the muB samples, which didn’t hinder downstream analysis. One library each of additional PCF11ΔPAS1 clones muC and muD has been sequenced as well.

#### Generation of zPCF11^null^ and zPCF11^ΔPAS1^ mutant fish

*zPCF11*^*null*^ and *zPCF11*^Δ*PAS1*^ mutant fish were generated by Cas9-mediated mutagenesis. To generate *zPCF11* knockout fish lacking zPCF11 protein, a guide RNA (sgRNA) targeting the first coding exon of the zPCF11 gene was generated according to published protocols by oligo annealing followed by T7 polymerase-driven *in vitro* transcription (gene-specific targeting oligo: zPCF11_ex1_gRNA; common gRNA oligo). To generate zebrafish lacking the conserved PAS1 in intron1 of *zPCF11*, a pool of four sgRNAs targeting intron1 sequences flanking the PAS1 (zPCF11_in1_gRNA1, zPCF11_in1_gRNA2, zPCF11_in1_gRNA3, zPCF11_in1_gRNA4) was generated in a similar manner. SgRNAs were co-injected together with Cas9 protein into the cell of one-cell stage TLAB embryos. Putative founder fish were outcrossed to TLAB wild-type fish. Founder fish carrying germline mutations in the first exon (primer: zPCF11_gt_F1 and zPCF11_gt_R1) or deletions in the first intron (primer: zPCF11_gt_F2 and zPCF11_gt_R2) of *zPCF11* were identified by size differences in the *zPCF11* PCR amplicons in pools of embryo progeny. Embryos from founder fish were raised to adulthood. Sanger sequencing of PCR products of genotyping reactions of adult fin-clips identified the nature of the mutations:

• *zPCF11*^*null*^: a 68-bp insertion in exon 1, which generates a frameshift mutation after amino acid 28 (H28), and introduces a premature STOP codon after an additional 16 amino acids (MSDDGAREDACREYQSSLEDLTFNSKPH - LVRYQLFQVDNGLSLF^∗^)
• *zPCF11*^Δ*PAS1*^: a 584-bp deletion in intron 1, which deletes the entire PAS1.

Homozygous *zPCF11*^*null*^ and *zPCF11*^Δ*PAS1*^ mutant embryos (*zPCF11*^*null*^*−/−* and *zPCF11*^Δ*PAS1*^*−/−)* were generated by incrossing heterozygous adult fish (*zPCF11*^*null*^*+/−* or *zPCF11*^Δ*PAS1*^*+/−)*. *zPCF11*^*null*^ mutant fish could only be maintained as heterozygotes due to embryonic lethality of *zPCF11*^*null*^*−/−* embryos.

#### Genotyping of zPCF11^null^ and zPCF11^ΔPAS1^ mutant fish

Genotyping of *zPCF11*^*null*^ fish (68-bp insertion) was performed by PCR amplification of exon 1 of the *zPCF11* gene (primers: zPCF11_gt_F1 and zPCF11_gt_R1). The PCR product size was analyzed by standard gel electrophoresis (wild-type allele: 200 bp, mutant allele: 268 bp).

Genotyping of *zPCF11*^Δ*PAS1*^ fish (584-bp deletion) was performed by two PCR reactions followed by standard gel electrophoresis. Using PCR reaction 1 (primers: zPCF11_gt_F2 and zPCF11_gt_R2), wild-type fish were reliably identified by the presence of a single 854-bp band. Heterozygous (PCR products of 270 bp and 854 bp) and homozygous (PCR product of 270 bp) fish were, however, not always reliably distinguished as the wild-type allele (upper 854-bp band) in the heterozygous fish was often only very weakly amplified. To identify homozygous fish definitively, PCR reaction 2 was performed using zPCF11_gt_F2 and reverse primer zPCF11_gt_R3, which binds in the intronic region that is deleted in *zPCF11*^Δ*PAS.1*^*.* This reaction only amplified the WT allele (369 bp), and homozygous mutant fish were therefore easily identified by a complete lack of PCR product.

#### Generation of zPCF11 full-length mRNA

The coding sequence of *zPCF11* was amplified by PCR from cDNA derived from zebrafish embryos (primers: zPCF11_F; zPCF11_R) and cloned by Gibson cloning into the BamHI/EcoRI-digested pCS2+ vector to generate P193: Sp6_zPCF11_SV40-3′UTR. The sequence of *zPCF11* was confirmed by Sanger sequencing. To generate *zPCF11* mRNA, P193 was linearized with NotI, and transcribed using the Sp6 mMessage Machine kit (Ambion). Functionality of the *zPCF11* mRNA was confirmed by the rescue of the fully penetrant brain necrosis phenotype of *zPCF11*^*null*^ −/− embryos by injection of 150 pg into 1-cell stage *zPCF11*^*null*^ −/− embryos.

#### Rescue and overexpression experiment of zPCF11^null^ mutant zebrafish

*zPCF11*^*null*^ heterozygous incrosses and wild-type embryos were injected with *zPCF11* mRNA (150 pg and 300 pg) and equimolar amounts of control mRNA (*GFP-Bouncer* (Herberg et al., 2018); 50 pg and 100 pg) through the chorion at the one-cell stage. Embryos were scored for morphological defects (e.g., head, tail, and heart defects) and brain necrosis at 1 day post fertilization (dpf) using a stereomicroscope (Zeiss).

For measurement of the length of the body axis, uninjected wild-type larvae and wild-type larvae that had been injected at the 1-cell stage with equimolar amounts of either *zPCF11* (150 pg or 300 pg) or control mRNA (*GFP-Bouncer* (Herberg et al., 2018); 50 pg or 100 pg) were dechorionated at 2 dpf, anesthetized with 0.1% tricaine (E10521, Sigma-Aldrich; 25x stock solution in dH_2_O, buffered to pH 7-7.5 with 1 M Tris pH 9.0) and imaged laterally using a standard stereomicroscope (Zeiss). Body axis length was measured from head to notochord tip using Fiji.

#### Phenotypic scoring of zPCF11^ΔPAS1^ mutant zebrafish

*zPCF11*^Δ*PAS1*^ homozygous mutant fish and wild-type control fish were scored for phenotypic defects and the presence or absence of a swim bladder at 5 days. To this end, larvae were anesthetized in 0.1% tricaine and phenotypically assessed using a standard stereomicroscope (Zeiss).

#### Immunostaining of zPCF11^ΔPAS1^ mutant zebrafish

Embryos were fixed at sphere stage in 3.7% PFA at 4°C overnight and washed in PBS-T (0.1% Tween20 in 1x PBS). Before immunostaining, embryos were permeabilized in 0.5% Triton X-100 in 1x PBS for 1 hr and re-fixed in 3.7% PFA for 20 min with subsequent washings in PBS-T. Embryos were blocked at 4°C overnight (in 20% NGS, 5% DMSO in PBS-T) and stained with a rabbit anti-PCF11 antibody (A303-705A, Bethyl Laboratories, used at 1:40) and a mouse anti-E-Cadherin antibody (610181, BD Biosciences, used at 1:400) at 4°C overnight. Secondary antibody staining was performed at 4°C overnight using goat anti-rabbit AlexaFluor-488 (A-11034, Thermo Fisher Scientific, used at 1:250) and goat anti-mouse AlexaFluor-546 (A-11003, Thermo Fisher Scientific, used at 1:250). DAPI staining was performed for visualize nuclei (incubation with 1x DAPI in PBST for 20 min at room temperature). Embryos were mounted in 1.5% low-melt agarose on a glass-bottom dish (81158, Ibidi) and imaged with an inverted LSM880 Axio Observer confocal microscope (Zeiss), using a 20x objective lens and 1.5x zoom.

#### 3′ mRNA-seq of zPCF11^null^ and zPCF11^ΔPAS1^ mutant zebrafish

Dechorionated embryos of heterozygous *Pcf11* mutant incrosses were cut in half with a razor blade at 19 hpf (*zPCF11*^*null*^) or 32 hpf (*zPCF11*^Δ*PAS1*^) and, and each head and tail was collected individually in PCR tubes. The anterior halves (heads) were lysed in 10 μl of TCL buffer with 1% beta-mercaptoethanol and flash-frozen on dry ice for subsequent use for RNA isolation and sequencing. The posterior halves (tails) were used for genotyping of each individual sample as described above. Between 3 and 6 individual samples (embryo heads) of each genotype (wild-type, heterozygous and homozygous) of *zPCF11*^*null*^ and *zPCF11*^Δ*PAS1*^ mutants were used for library preparation. RNA of selected samples was isolated and purified using Agencourt RNAClean XP magnetic beads (A63987, Beckman Coulter). Strand-specific libraries were generated using the QuantSeq 3′ mRNA Library Prep Kit FW (Lexogen) and used for 100-bp single-end sequencing on the Illumina HiSeq 2500.

### Quantification and Statistical Analysis

#### Human genomic annotation and analyzed gene sets

Hg19/GRCh37 was used as the reference genome. GENCODE release 19 was used for gene annotations: https://www.gencodegenes.org/. This annotation includes 57820 genes (20345 protein-coding, 37475 non-coding). For downstream analysis, we selected a subset of 11947 genes (9095 protein-coding, 2852 non-coding) that satisfied all of the following 3 criteria: 1) had at least one active PAS (see 3′ mRNA-seq analysis below for details); 2) did not overlap with another annotated gene on the same strand; 3) had a 3′ end isolated by at least 6 kb from the downstream annotated gene on the same strand. Those strand-specific isolation criteria allowed to unambiguously assign the directional RNA-seq signal (chrRNA-seq, mNET-seq and 3′ mRNA-seq) to the end of each gene, and also to compute distal alternative polyadenylation (APA) downstream of annotated gene ends (see below). 6 kb isolation was used because visual inspection of the data in genome browser revealed usage of cryptic non-annotated PASs used upon PCF11 depletion within this window. For meta-profiles and heatmaps, a subset of protein-coding genes longer that 5 kb was used (n = 8389), or a further subset of those as indicated in the figure legend. For calculation of distances between genes (Figures 4 and 7) the downstream distance from the gene’s 3′ end to any other annotated gene end (5′ or 3′) on either strand was computed.

#### ChIP-seq mapping, calibration, peak calling and enrichment definition

To allow for detection of proportional changes in global target enrichment we have added spike-in of *Drosophila melanogaster* chromatin and *Drosophila*-specific α-H2Av antibody to selected samples, as described in the experimental methods above, and as indicated in the GEO record. After quality control with FastQC the curated ChIP-seq reads were mapped with Bowtie2 using a genome index generated from combined *H. sapiens* hg19 and *D. melanogaster* dm6 genomes. Calculated density plots for distinct samples were normalized to both sequencing depth and the content of *Drosophila* reads (Egan et al., 2016). For PCF11 ChIP-seq samples, MACS2 was used to detect significant enrichments (broad peaks, q-value < 0.01). Peak calling was performed on combined reads from two biological replicates for each antibody. Only regions of overlap between the peaks called for the PCF11-Int and PCF11-Ct antibodies separately (peak intersection) were considered as PCF11-enriched. PCF11-enriched genes were further defined as a subset of the above described set of 11947 genes (active and separated within the same strand) which gene-body (TSS to PAS) or downstream region (PAS +5kb) overlapped with a PCF11-enriched region. PCF11-enriched genes were considered 3′ enriched if a PCF11-enriched region overlapped the region surrounding the PAS by −2kb to +5kb, independent of possible additional enrichment at the TSS or elsewhere on the gene. All other PCF11-enriched genes were categorized as TSS/gene body enriched.

#### mNET-seq mapping and analysis

Detailed computational mNET-seq workflow has been previously described (Nojima et al., 2016). In brief, reads in FASTQ files were trimmed with Cutadapt using following settings: -a TGGAATTCTCGG -A GATCGTCGGACT -e 0.05 -m 10–times 1 and mapped with STAR 2.5b to hg19. Last transcribed nucleotides positions from each read were retrieved using in house developed script based on R Bioconductor libraries. Those positions were further used to calculate genome-wide, sequencing depth normalized coverage utilized for downstream analysis and visualization.

#### Chromatin RNA-seq mapping and analysis

After quality control with FastQC curated reads were mapped with STAR 2.5b aligner to hg19 (index generated with GRCh37.p13 assembly and gencode.v19.annotation.gtf annotation file). Genomic coverage was normalized to sequencing depth for downstream analysis and visualization.

#### 3′ mRNA-seq mapping

3′ mRNA-seq data were mapped according to the guidelines of QuantSeq library kit manufacturer (Lexogen). Unaligned bam files from HiSeq2500 were converted to FASTQ files with bam2fastx (TopHat 2 component). After overview with FastQC reads were trimmed with BBtools (https://sourceforge.net/projects/bbmap/) script bbduk using following settings: k = 13 ktrim = r useshortkmers = t mink = 5 qtrim = r trimq = 10 minlength = 20. After trimming control with FastQC curated reads were mapped with STAR2.5b aligner to hg19 (index generated with the GRCh37.p13 assembly and gencode.v19.annotation.gtf annotation file).

For PAS calls and APA analysis (below) we have adapted previously published work flows (Derti et al., 2012, Fontes et al., 2017, Rot et al., 2017).

#### Calling polyadenylation sites (PAS)

1. Aligned 3′ mRNA-seq reads were filtered to remove false positives due to internal priming of the QuantSeq assay on genome-encoded poly(A) stretches. To do this, first a crude genomic mask was generated that contained all loci harboring 6 or more consecutive A bases as well as any 10 nucleotide windows containing more than 6 A bases. For genes expressed from the reverse strand, an analogous T-rich mask was generated. Those crude masks were then corrected to allow for detection of genuine PAS falling in A/T-rich regions by strand-specifically unmasking 20 nucleotide intervals centered at GENCODE 3′ gene ends as well as previously experimentally validated PAS sites detected in all human datasets from (Derti et al., 2012). 3′ mRNA-seq reads falling into those refined strand-specific masks were then removed, and the filtered reads reduced to the most distal nucleotide (3′ end nucleotide).
2. PAS calling: based on filtered PAS (appropriate 3′ mRNA-seq read ends) strand-specific, sequencing depth corrected, genome-wide density profiles were computed. Density plots from all human 3′mRNA-seq samples were summed up, separately for each strand. PAS events were detected in the following way: First, all signals within 30 nt windows were summed up and windows with values above 30 were merged. Further local signal maxima within obtained intervals were found and new 30 nt intervals centered at those maxima were generated. This procedure could in some cases lead to generation of overlapping intervals, to avoid this it was repeated again resulting in the set of PAS which were used for both analysis of alternative polyadenylation sites usage (APA) and differential expression analysis.
3. This set of active PAS was then used to count the PAS usage in every sample. The results were sequencing depth-normalized for visualization. APA (DEXseq) and DE analysis were performed on non-normalized reads as the methods used rely on own normalization procedures.

#### Quantification of alternative polyadenylation (APA)

To quantify APA, DEXseq (https://bioconductor.org/packages/DEXSeq) was employed (following previously published workflows for APA analysis (Rot et al., 2017, Fontes et al., 2017). Genes from the analysis set which had at least two alternative active PAS (7718 genes) were subject to differential PAS usage quantification with DEXSeq. Gene coordinates were extended by 6kb downstream of the annotated 3′ end to allow for detection of distal APA beyond annotated gene ends. 4 biological repeats for PCF11 knock-down and 3 biological repeats for PCF11 PAS1 deletion were assayed. Genes where no PAS usage changed significantly between control and treated conditions (DEXseq p-adjusted > = 0.05) were categorized as APA no shift. To determine the direction of APA shift in genes with altered APA, two most statistically differentially used PAS were selected (DEXseq p-adjusted < 0.05), or in the case of genes where only one PAS was significantly altered it was compared to the most frequently used other PAS. If the ratio of the distal to proximal site usage was higher in the treated than in the control cells, the shift was classified as distal, in the opposite situation as proximal (Rot et al., 2017).

#### Analysis of differential gene expression (DE)

Differential expression analysis was performed with DESeq2 (https://doi.org/10.18129/B9.bioc.DESeq2), a well established package from the R Bioconductor project, using the unpaired experimental design. Gene expression was defined as a sum of 3′ mRNA-seq read counts falling into called PAS within gene coordinates extended by 6 kb on 3′ end. Genes with p-adjusted < 0.05 were classified as differentially expressed.

#### Identification of PCF11-attenuated genes

First, protein coding genes from the gene analysis set, larger than 5 kb, and upregulated upon PCF11 knock-down (log_2_FC > 0, DESeq2 padj < 0.05) were identified. For premature termination definition, their gene bodies were divided in 25 equal bins. The mean T4ph mNET-seq signal was computed in the first 20 bins and genes where the T4ph mNET-seq signal ratio between siLUC/siPCF11 > 2 were considered prematurely terminated (n = 166). For premature CPA definition, we selected upregulated genes containing multiple APA sites, in which at least one PAS different from the major PAS decreased significantly in siPCF11 versus siLUC cells (DEX-seq padj < 0.1; n = 107 genes). The sum of those gene sets was 218 genes, 55 genes overlapped between the categories. Identified putatively PCF11-attenuated genes are listed in Table S2. GO analysis was performed using GeneCodis3 software as implemented on the webpage (http://genecodis.cnb.csic.es) using GO Biological Process annotation, padj < 0.01 and gene number > 2. To ensure robustness, the GO analysis was repeated on more stringently defined attenuated gene sets, e.g., on genes where the PAS decreasing in siPCF11 conditions was > 2kb upstream of the major PAS, and similar results were obtained i.e., regulation of transcription, DNA-dependent; gene expression; and RNA processing-related categories were in each case the most significantly enriched processes.

#### Metagene profiles and heatmaps

Metagene profiles and heatmaps were generated in R, based on GENCODE annotation. Enrichment density plots were binned into 50 bp intervals and represent an average of biological replicates. Maximum PAS signal in our datasets was taken as the gene’s 3′ end coordinate. ChIP-seq and mNET-seq data were binned into 50 bp bins. Mean read counts in bins ± 5kbp from the TSS and −5kb/+10kb from the maximum PAS were extracted for every gene from the analysis gene set limited to protein-coding genes longer than 5 kbp (n = 8389). Gene body (GB) of each of gene was divided into 25 equally-sized GB bins and the mean read counts were calculated for each bin. Sequence of bins from genes transcribed from the negative DNA strand was reversed. Finally metagene profiles for distinct genes were assembled by combining respective 5′ end profiles (TSS −5/+1.5 kbp), gene body profiles (GB bins 4:22) and 3′ end profiles (PAS −1.5/+10 kbp). For heatmaps metagene profiles were arranged into matrix sorted based on the signal intensity and visualized with the R ggplot2 package. Mean matagene profiles were calculated as matrices column means.

#### Grouping of genes into proximal and distal major PAS categories

For analysis of association of T4ph mNET-seq signal with proximal and distal APA sites, we selected protein-coding APA genes where the signal of the two strongest PASs differed no more than 2-fold, and that were separated by at least 2 kb, which resulted in a group of 938 genes. The reason for selecting genes with at least two PASs of similar strength was that in case of genes with a very dominant PAS a lack of mNET-seq signal downstream of the minor PASs could be due to a detection limit. The 2kb distance criterion was used, because on average the strongest T4ph mNET-seq signal in control cells is observed within 2kb from the major PAS (see Figure 1C), therefore this distance generally allows to separate T4ph mNET-seq signals from alternative PASs. Those 938 genes were further divided into two groups – one where the major PAS was proximal (PAS1 signal > PAS2 signal, n = 413) and second where the major PAS was distal (PAS1 signal ≤ PAS2 signal, n = 525).

#### Zebrafish genomic annotation and 3′ mRNA-seq data analysis

The analyses of zebrafish 3′ mRNA-seq data (read mapping, APA and DE analysis) were all done using the same workflow as established for the human datasets, with the following adjustments. GRCz10/danRer10 was used as the reference zebrafish genome together with the corresponding ENSEMBL annotation. The read filtering mask used the annotated ENSEMBL 3′ ends. For the *zPCF11*^*ΔPAS1*^ mutant the *zPCF11* gene PAS category was manually corrected to APA proximal, as *zPCF11* PAS1 was deleted genetically. For attenuation detection in the zebrafish datasets, similarly to human datasets, we selected genes > 5kb long that were significantly upregulated in *zPCF11*^*null*^ −/− versus +/+ (DESeq2 padj < 0.05). As discussed in the manuscript, using DEXseq we observed much less significantly altered PAS in zebrafish compared to human (possibly due to high variability of heterogenous embryo tissue samples). For this reason, to detect changes in CPA we employed a criterion of minimum 2-fold decreasing average PAS signal in *zPCF11*^*null*^ −/− versus +/+ sample replicates, instead of DEXseq significance used for human samples.

#### Data mining and re-analysis of published datasets

Data in Figures S4E, S4F, and S5A and Table S1 have been extracted from global quantitative proteomics datasets (Nagaraj et al., 2011, Wiśniewski et al., 2015b, Wiśniewski et al., 2015a, Wiśniewski et al., 2016) from respective supplemental tables.

In Figure 5B, the CPA complex subunits mRNA stability and abundance (as determined by BRICseq) was extracted from table S1 in (Tani et al., 2012).

In Figure S5C, 3′READS dataset (Li et al., 2015) was re-analyzed using the read counts provided by authors in processed table attached to the GEO dataset GSE62001. PAS1 usage was defined as *PCF11* PAS1 reads relative to total *PCF11* 3′READS reads in each sample. This value was then divided by the PAS1 usage of the corresponding siCtrl sample to get the relative PAS1 usage values plotted.

In Figure S5I, PCF11 PAS usage in human tissue was extracted from APASdb (You et al., 2015), which collects datasets specifically profiled for polyadenylation sites using the SAPAS method. Plotted are the processed data as accessed from the database at http://genome.bucm.edu.cn/utr/. For human tissues analysis, tissues were ordered according increasing numbers of PCF11 total PASs mapped i.e., increasing number of transcripts. Colors indicate PCF11 PAS1 usage levels: white/no corresponds to no sequencing counts, yellow/low to 4-10 sequencing counts, orange/medium to 10-30 counts and red/high to > 30 counts.

In Figures S6A and S6E, plotted are zebrafish RNA-seq and 3P-seq data from (Ulitsky et al., 2011, Pauli et al., 2012, Herberg et al., 2018) derived from GEO series GSE32880, GSE32900 and GSE111882.

In Figure 7F, shown are Pol II S2ph ChIP-seq data from (Fong et al., 2017) and nucleoplasmic RNA-seq data from (Nojima et al., 2015). Bigwig files downloaded from GEO series GSE97827 and GSE60358 respectively were directly uploaded to UCSC genome browser to create the genomic profiles.

#### Statistical analysis

Statistical details of experiments are described in the figure legends. n corresponds to the number of genes assayed in a given genomic analysis, or to the number of independent experiments for all other analyses. In all boxplot graphs the bottom and top of the box represent the 25^th^ (Q_1) and 75^th^ (Q_3) percentile respectively and the thick horizontal line the median. The whiskers are defined as:

upper whisker = min(max(x), Q_3 + 1.5 ∗ IQR)

lower whisker = max(min(x), Q_1 – 1.5 ∗ IQR)

where IQR = Q_3 – Q_1, the box length. Results presented in all bar charts (mainly western blot quantifications) are expressed as mean values with error bars indicating standard deviation (SD). Statistical significance was analyzed using a Mann-Whitney test or unpaired two-tailed t test as appropriate, indicated in figure legend. ^∗^ p ≤ 0.05, ^∗∗^ p ≤ 0.01 and ^∗∗∗^ p ≤ 0.001 were considered significant.

### Data and Software Availability

The accession number for all NGS datasets (mNET-seq, chromatin RNA-seq, 3′mRNA-seq and ChIP-seq) generated in this paper is GEO: GSE123105. Original images of western blot, gel and immunofluorescent staining assays are available at Mendeley Data (https://doi.org/10.17632/rmjm32hd6n.1). Software used in this work is publicly available under web links indicated in the Key Resources Table.

## Supplemental Information

Document S1. Figures S1–S7

Table S1. Number of Protein Molecules per Cell of CPA Complex Subunits and CID-Containing Proteins, Related to Figure 5

Table S2. List of Human Genes Attenuated by PCF11, Related to Figure 7

Table S3. List of Primers Used in This Study, Related to STAR Methods Further processed data tables uploaded with the genomic datasets to GEO.

Document S2. Article plus Supplemental Information
