# Supplementary material for: Selective Roles of Vertebrate PCF11 in Premature and Full-Length Transcript Termination
Source: Mol Cell. 2019 Apr 4;74(1):158–172.e9. doi: 10.1016/j.molcel.2019.01.027 (PMC6458999; doi:10.1016/j.molcel.2019.01.027)
Supplement: Document S1. Figures S1–S7 [file mmc1.pdf]

Molecular Cell, Volume *74*

Supplemental Information

Selective Roles of Vertebrate PCF11 in

Premature and Full-Length Transcript Termination

Kinga Kamieniarz-Gdula, Michal R. Gdula, Karin Panser, Takayuki Nojima, Joan Monks, Jacek R. Wiśniewski, Joey Riepsaame, Neil Brockdorff, Andrea Pauli, and Nick J. Proudfoot

Figure S1

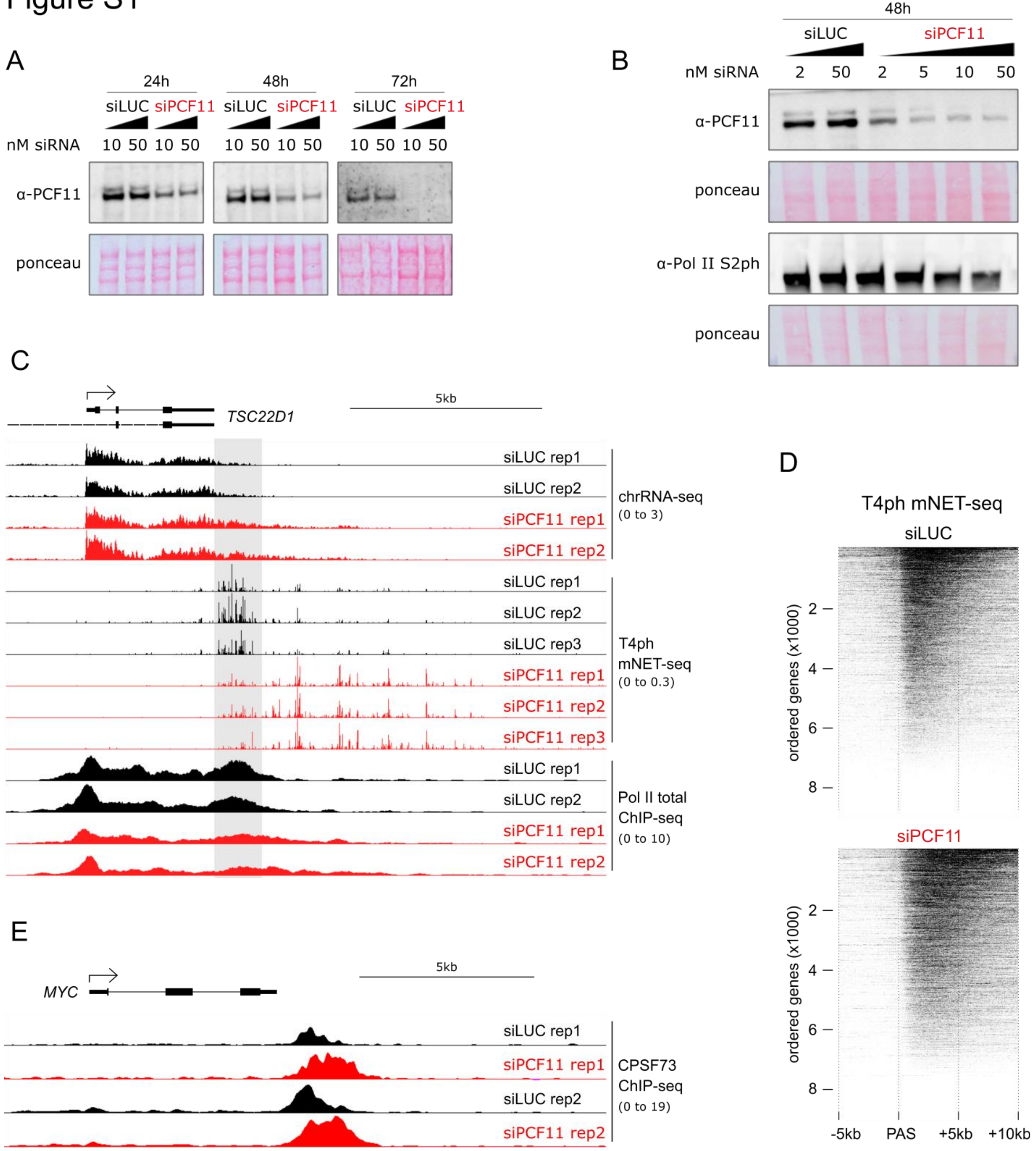

Figure S1. Related to Figure 1

(A and B) Western blot (WB) showing efficiency of PCF11 depletion. Cells were transfected with siRNA against luciferase (siLUC, control cells; black) or siRNA against PCF11 (siPCF11, red). Increasing concentrations of siRNAs are indicated by black triangles. The two WB bands result from PCF11 protein isoforms, as verified by Mass Spectrometry. (A) Time course of siRNA treatment. (B) Range of siRNA concentrations applied for 48 hr. Note that at high concentration of siPCF11 a decrease in RNA Pol II S2ph signal can be observed.

(C) Genomic profiles showing termination defects in *TSC22D1* upon PCF11 depletion (siPCF11, red) (biological repeats of Figure 1A). For chrRNA-seq and mNET-seq only the sense strand is shown. Grey shading highlights the termination window in control cells (siLUC, black). In all genomic profiles: numbers in brackets indicate the viewing range (rpm).

(D) Heatmaps showing T4ph mNET-seq profiles across individual protein-coding genes ordered based on their T4ph mNET-seq levels (n=8389, gene set as in Figure 1B-D). Top: control cells, bottom: PCF11 depleted cells.

(E) Genomic profile of CPSF73 ChIP-seq binding to *MYC* ±siPCF11.

Figure S2

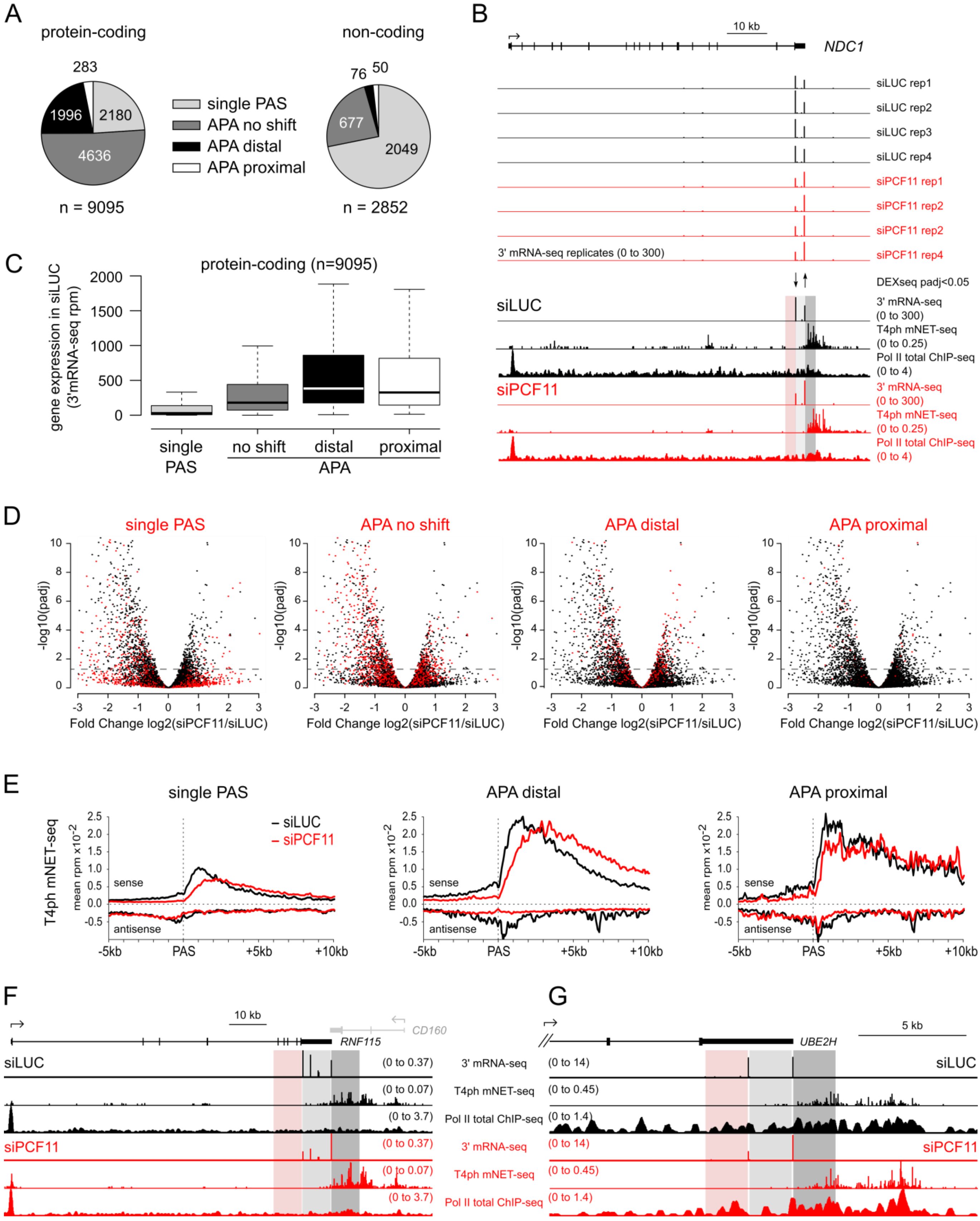

Figure S2. Related to Figure 2

(A) Pie chart of PAS usage upon PCF11 depletion in pc-genes (left) and nc-genes (right): subsets of analysis shown in Figure 2B.

(B) Genomic snapshot of the full *NDC1* locus. Top: biological replicates of 3'mRNA-seq ±siPCF11. Arrows indicate significantly changed PAS usage ±siPCF11 (DEXseq p-adjusted < 0.05). Bottom: average 3' mRNA-seq, T4ph mNET-seq and Pol II ChIP-seq signal. Red shading highlights the region 2kb upstream of the proximal PAS, light grey the ~2kb region between proximal and distal PAS and dark grey the region 2kb downstream of the distal PAS.

(C) Box plot showing expression levels (measured by 3' mRNA-seq) of pc-genes split into the four PAS usage categories. Here and in all boxplot figures, the thick horizontal line marks to the median, and the upper and lower limits of the box the first and third quartile.

(D) Volcano plots showing differential expression of pc-genes (n=9095) - negative fold change values: genes down-regulated, positive values: genes up-regulated upon PCF11 depletion. Genes from each PAS usage category (top label) are depicted in red, genes from all other categories shown in black as reference. Genes above the horizontal dashed line have significantly changed expression upon PCF11 depletion (DEseq p-adjusted<0.05 corresponding to -log10(padj)>1.3 in the graph).
(E) Metagene analysis of T4ph mNET-seq signal ±siPCF11 around the major PASs of pc-genes in the indicated PAS usage categories. APA no shift category is shown in Figure 2E.
(F-G) Genomic profiles of *RNF115* and *UBE2H*. The shadings highlight the regions: upstream of the proximal PAS (red), in between proximal and distal PASs (light grey) and downstream of the distal PAS (dark grey). The highlighted regions correspond to ~7.5kb for *RNF115* and ~2kb for *UBE2H*.

Figure S3

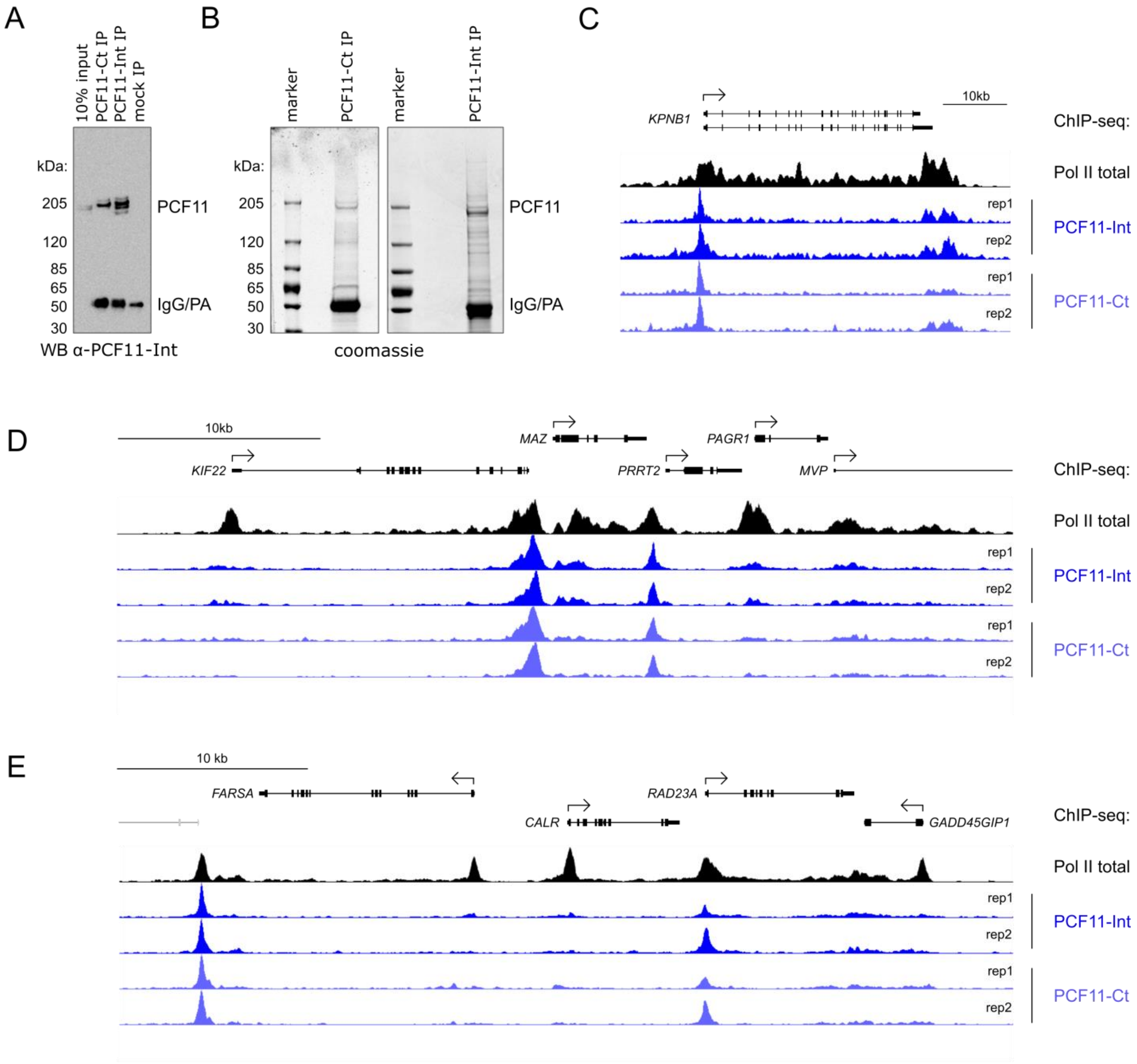

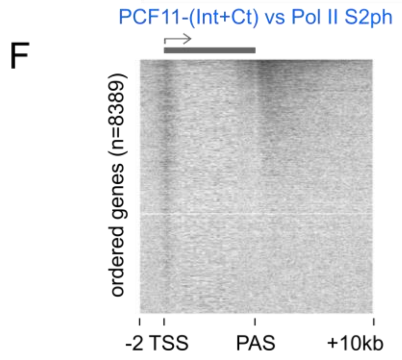

G

alternative 3' processing mechanism mediated by:	example genes:
U7 snRNP	HIST1H1C, HIST1H3B
RNase P	MALAT1, NEAT1
integrator	RNU1-1, RNVU1-14
microprocessor	MIR17HG, MIRLET7I

Figure S3. Related to Figure 3.

(A) Western blot showing efficiency of PCF11 IP. PCF11 was immunoprecipitated using either α-PCF11-Ct or α-PCF11-Int antibodies. The membrane was probed with α-PCF11-Int.

(B) Coomassie staining of proteins immunoprecipitated with α-PCF11-Ct (left) and α-PCF11-Int antibodies (right). Even under mild conditions (150mM NaCl, 0.5% NP40), the major protein bands just below the 205kDa marker correspond to PCF11 isoforms, as verified by Mass Spectrometry. (A and B) IgG/PA denotes the migration size of immunoglobulin heavy chain (IgG) and protein A (PA).

(C-E) Genomic profiles of PCF11 ChIP-seq binding in the *KPNB1, MAZ*, and *CALR* loci showing 2 biological ChIP replicates using both antibodies (4 ChIP-seq samples total). ChIP-seq for Pol II is shown as reference. Viewing range was autoscaled to maximum signal. Grey outline in (E) corresponds to inactive *SYCE2*.

(F) Heatmap of PCF11-(Int+Ct) ChIP-seq signal normalized to Pol II S2ph across protein-coding genes ranked from highest to lowest PCF11 signal.

(G) Table of alternative 3' processing mechanism used by non-canonical (non-CPA-dependent) PCF11 target genes.

Figure S4

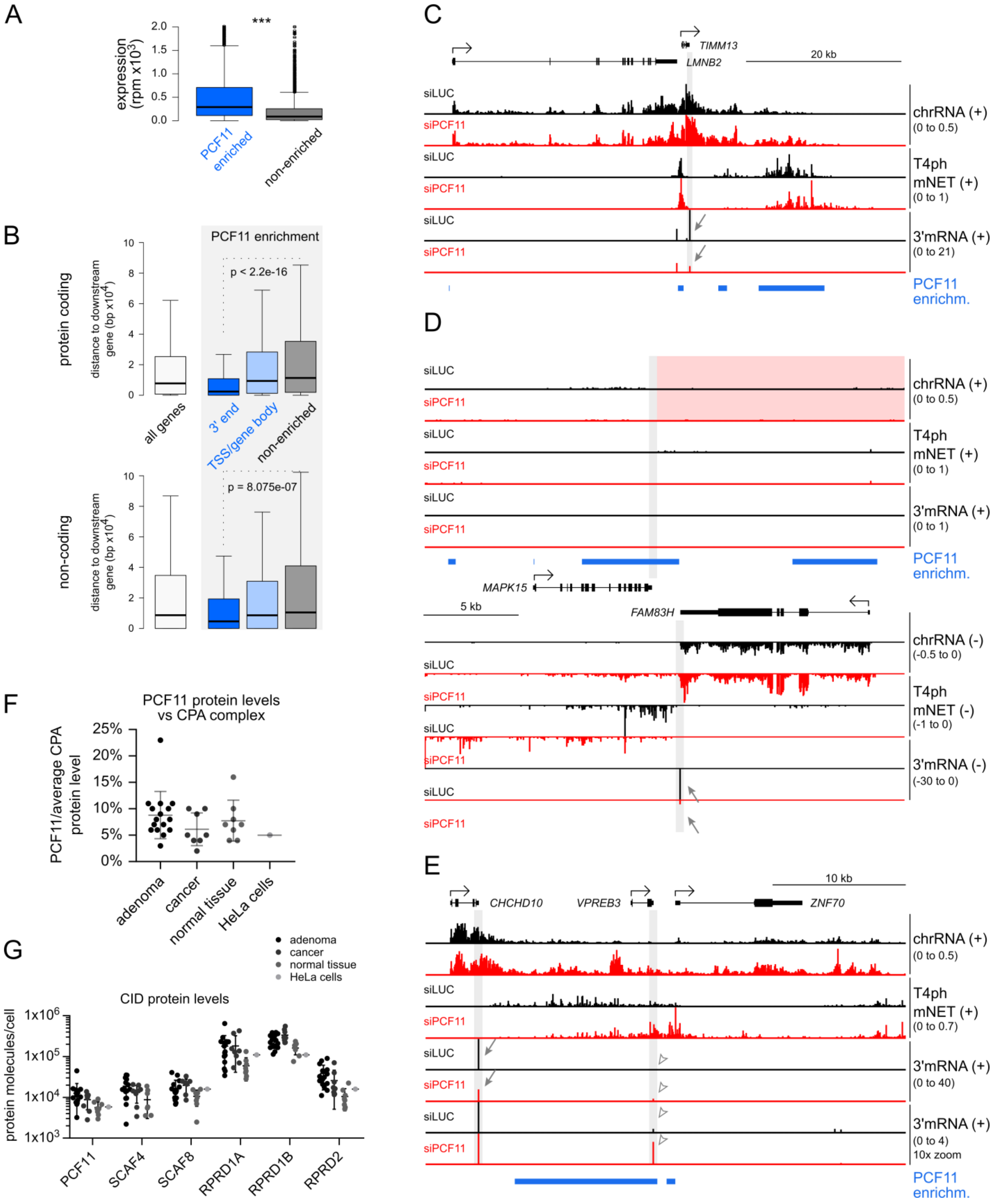

Figure S4. Related to Figure 4 and 5

(A) Comparison of gene expression levels of PCF11 enriched and non-enriched genes based on 3' mRNA-seq reads.
(B) Boxplots showing the distances between gene PAS and nearest gene downstream. Top: protein coding genes, bottom: non-coding genes. (A and B) Statistical significance was determined using Mann-Whitney test.
(C-E) Genomic profiles of the indicated loci. Grey shading highlights the 3' ends of relevant genes. Red shading highlights the lack of detectable read-through from *MAPK15* into the highly downregulated *FAM83H*. Blue bars: PCF11 enriched regions; arrows: gene downregulation measured by 3' mRNA-seq; empty arrowheads: upregulation of *VPREB3* due to read-through transcription from *CHCHD10*.
(F) Quantification of PCF11 protein molecules per cell relative to the average copy number of other CPA complex subunits in biopsies from colorectal adenoma (n=16), cancer (n=8) and normal tissue (n=8), as well as in HeLa cell culture (n=1) based on global quantitative proteomics. PCF11 levels are on average 10-20 fold lower (5-9%) compared to other CPA complex subunits.
(G) Scatter plot of copy numbers of human CID-containing proteins in the same samples as (F). SCAF4 hasn't been assayed in HeLa cells. (F and G) Data from (Nagaraj et al., 2011; Wiśniewski et al., 2015). Horizontal lines indicate the mean and SD.

Figure S5

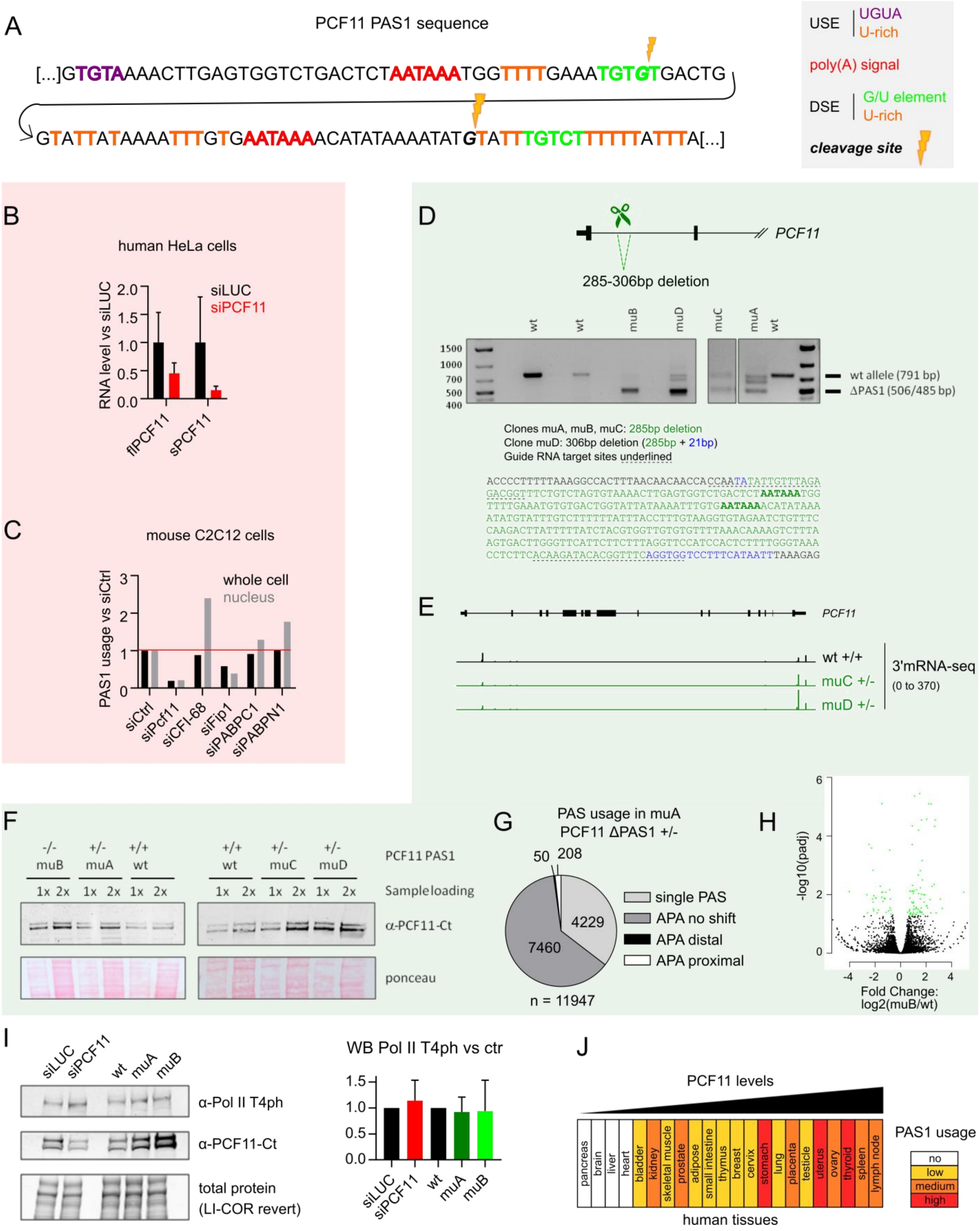

Figure S5. Related to Figure 5.

(A) DNA sequence of *PCF11* PAS1. Indicated are the two tandem AATAAA hexamers (red), as well as additional upstream and downstream elements (USE and DSE) forming the poly(A) signal. Two alternative cleavage sites are indicated in *italics* and by the yellow lightning bolt. The second cleavage site is more frequently used.

(B) RNA levels of full-length *PCF11* mRNA (*flPCF11*) and the short *PCF11* isoform (*sPCF11*) in human HeLa cells ±siPCF11 based on 3'mRNA-seq (error bars correspond to SD, n=4). Plotted are values relative to the average levels in siLUC.

(C) *PCF11* PAS1 usage in mouse C2C12 cells depleted for indicated CPA factors, relative to control cells. Values are based on PAS sequencing using 3'READS method from (Li et al., 2015). Sequenced RNA was extracted either from whole cells (black) or isolated nuclei (grey).

(D) *PCF11* PAS1 deletion using CRISPR/Cas9. (Top) schematic of the deletion, (middle) PCR analysis of the mutant clones showing degree of PAS1 deletion. Sizes of the wt allele and sequenced deletion alleles are indicated. Only clone muB shows a complete deletion of PAS1. Clones muA, muC and muD are heterozygous, with muD showing the highest proportion of deleted alleles. (Bottom) nucleotides deleted (green and blue) in the four mutant clones based on Sanger sequencing of the PCR products. Underlined nucleotides are CRISPR/Cas9 guide RNA targets, the two tandem poly(A) hexamer signals indicated in bold.

(E) Profile of 3'mRNA-seq on the *PCF11* gene in wt cells and clones muC and muD.

(F) WB of PCF11 protein levels in wt cells and *PCF11* PAS1 mutant clones muA-muD. Each sample was loaded at two concentrations, as indicated.

(G) Pie chart of genome-wide PAS usage and APA occurrence in *PCF11ΔPAS1* clone muA vs wt cells (compare with Figures 5I and 2B).

(H) Volcano plot showing differential expression between wt and *PCF11ΔPAS1* clone muB cells. Genes indicated by green dots have significantly changed expression upon PCF11 depletion (DEseq p-adjusted<0.05). 2 times more genes are significantly upregulated than downregulated in *PCF11ΔPAS1* clone muB vs wt cells.

(I) WB analysis of Pol II T4ph levels in cells where PCF11 is downregulated (siPCF11) or upregulated (*PCF11ΔPAS1*: muA and muB). Left: representative WB, right: quantification of WB experiments, average values relative to control, error bars correspond to SD (n=6 for PCF11 depletion; n=4 for *PCF11ΔPAS1*)

(J) *PCF11* PAS1 usage in 22 human tissues. The tissues were ranked according to increasing *PCF11* mRNA levels. Colours indicate *PCF11* PAS1 usage levels: white, no sequencing counts; yellow, 4-10 sequencing counts; orange, 10-30 counts and red, >30 counts. Data extracted from APASdb (You et al., 2015).

Figure S6

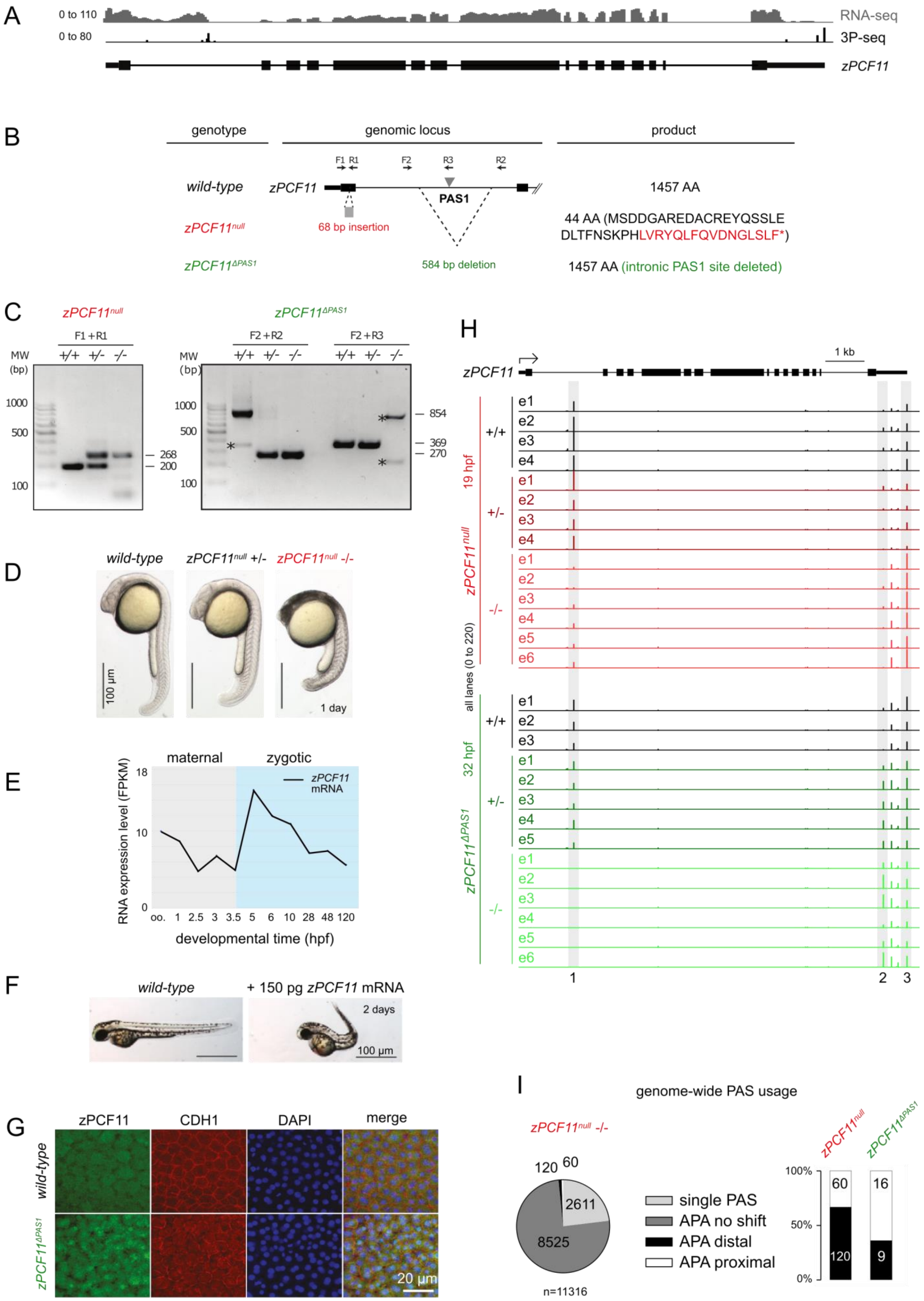

A
0 to 110
RNA-seq
0 to 80
3P-seq
zPCF11
B
genotype
genomic locus
product
F1 R1 F2 R3 R2
wild-type
zPCF11
PAS1
1457 AA
zPCF11null
68 bp insertion
44 AA (MSDDGAREDACREYQSSLE DLTFNSKPHLVRYQLFQVDNGLSLF*)
zPCF11ΔPAS1
584 bp deletion
1457 AA (intronic PAS1 site deleted)
C
zPCF11null
zPCF11ΔPAS1
F1 + R1
F2 + R2
F2 + R3
MW (bp)
+/+ +/- -/-
1000
500
100
268
200
854
369
270
D
wild-type
zPCF11null +/-
zPCF11null -/-
100 µm
1 day
E
maternal
zygotic
zPCF11 mRNA
RNA expression level (FPKM)
18
10
0
oo. 1 2.5 3 3.5 5 6 10 28 48 120
developmental time (hpf)
F
wild-type
+ 150 pg zPCF11 mRNA
2 days
100 µm
G
zPCF11
CDH1
DAPI
merge
wild-type
zPCF11ΔPAS1
20 µm
H
1 kb
zPCF11
19 hpf
zPCF11null
all lanes (0 to 220)
32 hpf
zPCF11ΔPAS1
+/+
+/-
-/-
e1
e2
e3
e4
e5
e6
1
2
3
I
genome-wide PAS usage
zPCF11null -/-
120
60
2611
8525
n=11316
single PAS
APA no shift
APA distal
APA proximal
zPCF11null
zPCF11ΔPAS1
100%
50%
0%
60
120
16
9

Figure S6. Related to Figure 6.
(A) Genomic locus of z*PCF11*. Shown is signal of RNA-seq from ovary (GSE111882, Herberg et al., 2018) and 3P-seq from adult fish (GSE32880, Ulitsky et al., 2011).
(B) Overview of zebrafish *zPCF11*^{null} and *zPCF11*$^{\Delta PAS1}$ mutants. The positions of primer sequences used for genotyping and predicted protein products of the wild-type and mutant forms are indicated. Only first two exons of *zPCF11* are depicted.
(C) Genotyping PCR of wild-type, *zPCF11*^{null} and *zPCF11*$^{\Delta PAS1}$ mutant embryos. Primer locations are shown in Figure S6B. Bands indicated by asterisks are background.
(D) *zPCF11*^{null}-/- mutant larvae show severe brain necrosis at 1 day.
(E) *zPCF11* mRNA is maternally provided and zygotically expressed. Data from GSE32900 (Pauli et al., 2012) and GSE111882 (Herberg et al., 2018), oo. = oocyte.
(F) Example images of wild-type or *zPCF11* overexpressing larvae at 2 days.
(G) Immunofluorescence of wild-type and *zPCF11*$^{\Delta PAS1}$-/- embryos at 5 hours post fertilization. Note the increased PCF11 signal in *zPCF11*$^{\Delta PAS1}$-/- embryos.
(H) Profile of 3' mRNA-seq reads from individual zebrafish embryo heads (e1-e6) of the indicated genotypes at the *zPCF11* locus. Grey shadings indicate PASs undergoing APA.
(I) (Left) PAS usage in *zPCF11*^{null}-/- mutants vs wild-type. Categories identical to Figure 2A-B. (Right) Significant APA changes in *zPCF11*^{null}-/- and *zPCF11*$^{\Delta PAS1}$-/- mutants (DEXseq padj < 0.05).

Figure S7

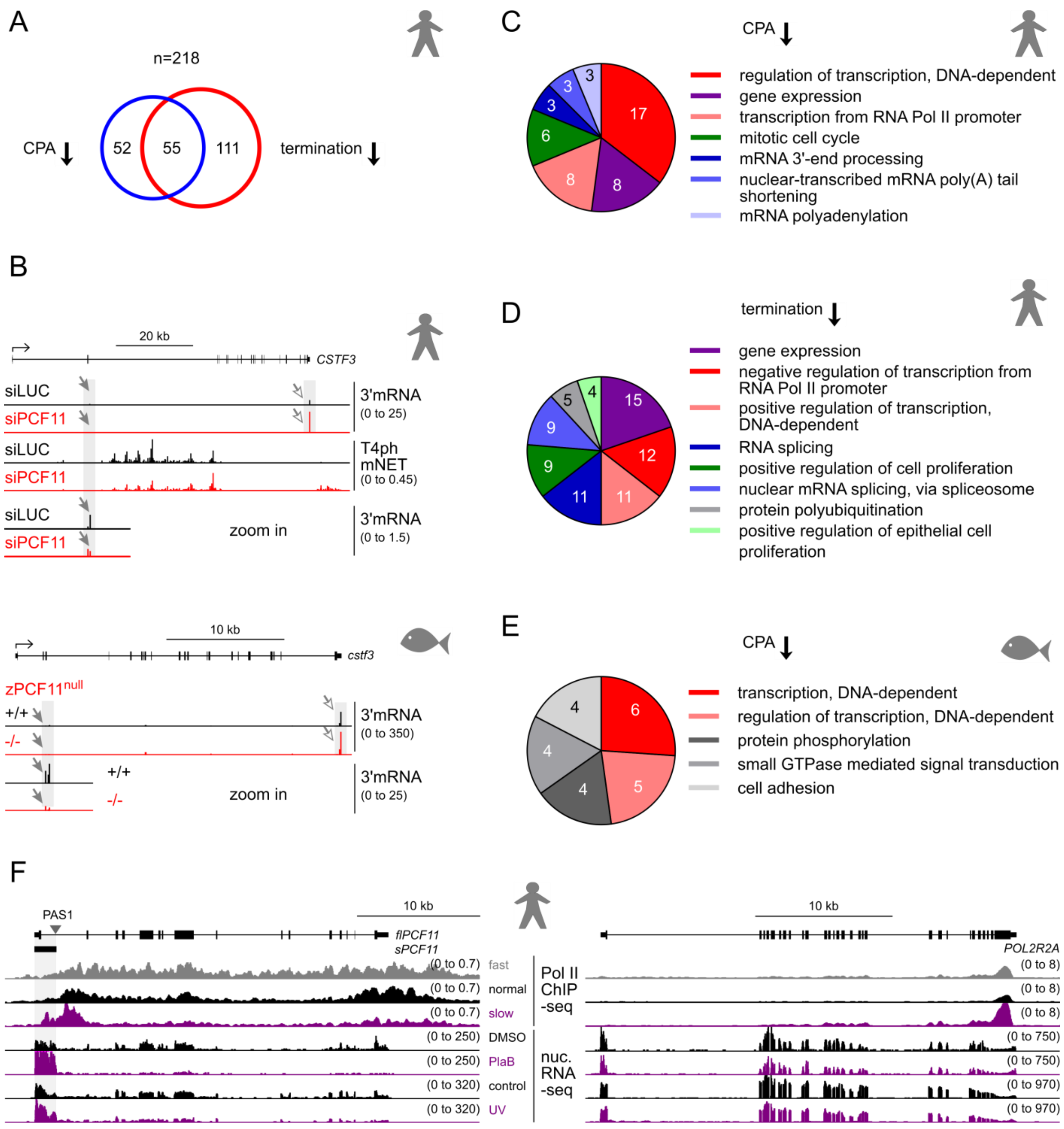

Figure S7. Related to Figure 7 and Discussion
(A) Number of genes and overlap between genes undergoing premature CPA (left) and premature termination (right) in human cells.
(B) Genomic profiles of *CSTF3* ±PCF11 for human cells (top) and zebrafish embryos (bottom). Grey shading and arrows highlight distal APA in PCF11 depleted conditions (grey arrowheads: decreased intragenic PAS usage, white arrowheads: increased 3' UTR PAS usage).
(C to E) Enrichment analysis of GO Biological Process for genes undergoing premature CPA (C) and premature termination (D) in human cells, and genes undergoing premature CPA in zebrafish (E). Parameters used as in Figure 7B. Red shades; genes related to transcription; blue shades, genes related to RNA processing.
(F) Genomic profiles of the *PCF11* (left) and *POLR2A* (right) showing the effect of various treatments on the transcription of these two genes. Top: Pol II S2ph ChIP-seq in cells expressing kinetic mutants of Pol II: fast (E1126G), normal (wild-type) and slow (R749H). Data from (Fong et al., 2017). Bottom: RNA-seq of the nucleoplasmic fraction in control cells, cells treated with pladienolide B (PlaB, splicing inhibitor) for 4 hours (Nojima et al., 2015), or cells harvested 2 hours after UV treatment with 20mJ/cm^2 (UV, T. Nojima). Grey shading highlights the coordinates of the *sPCF11* isoform ending with PAS1. Note that the slow Pol II mutant, splicing inhibition, and UV treatment (purple lanes) all lead to preferential *sPCF11* transcription and concomitant *flPCF11* downregulation.
